# Auxetic piezoelectric effect in heterostructures

**DOI:** 10.1038/s41563-023-01736-5

**Published:** 2023-11-30

**Authors:** Ming-Min Yang, Tian-Yuan Zhu, Arne Benjamin Renz, He-Meng Sun, Shi Liu, Peter Michael Gammon, Marin Alexe

**Affiliations:** 1 Department of Physics, The University of Warwick, Coventry, UK; 2 Hefei National Laboratory, Hefei, China; 3 School of Emerging Technology, The University of Science and Technology of China, Hefei, China; 4 Key Laboratory for Quantum Materials of Zhejiang Province, Department of Physics, School of Science and Research Center for Industries of the Future, Westlake University, Hangzhou, China; 5 Institute of Natural Sciences, Westlake Institute for Advanced Study, Hangzhou, China; 6 School of Engineering, The University of Warwick, Coventry, UK

**Keywords:** Ferroelectrics and multiferroics, Electronic properties and materials

## Abstract

Inherent symmetry breaking at the interface has been fundamental to a myriad of physical effects and functionalities, such as efficient spin–charge interconversion, exotic magnetic structures and an emergent bulk photovoltaic effect. It has recently been demonstrated that interface asymmetry can induce sizable piezoelectric effects in heterostructures, even those consisting of centrosymmetric semiconductors, which provides flexibility to develop and optimize electromechanical coupling phenomena. Here, by targeted engineering of the interface symmetry, we achieve piezoelectric phenomena behaving as the electrical analogue of the negative Poisson’s ratio. This effect, termed the auxetic piezoelectric effect, exhibits the same sign for the longitudinal (*d*_33_) and transverse (*d*_31_, *d*_32_) piezoelectric coefficients, enabling a simultaneous contraction or expansion in all directions under an external electrical stimulus. The signs of the transverse coefficients can be further tuned via in-plane symmetry anisotropy. The effects exist in a wide range of material systems and exhibit substantial coefficients, indicating potential implications for all-semiconductor actuator, sensor and filter applications.

## Abstract (web summary)

Piezoelectrics have longitudinal and transverse piezoelectric coefficients that are opposite in sign. Here, by tuning the interface inversion asymmetry in heterostructures, auxetic systems with positive longitudinal and transverse coefficients are realized, with expansion or contraction along all directions in an electric field.

## Main

The piezoelectric effect has been discovered and exploited in a wide range of materials with innate bulk non-centrosymmetric structures^1–5^, such as ferroelectric oxides possessing morphotropic phase boundaries^5^, polymers^6^, two-dimensional layered materials^7^ and even bones^8^. Despite these materials’ diverse symmetries, structures, chemical natures and general material properties, the signs of their piezoelectric coefficients can hardly be modulated. The longitudinal (*d*_33_) and the transverse (*d*_31_, *d*_32_) coefficients of the piezoelectric materials experimentally explored so far are of opposite signs (Supplementary Table 1). For example, the PbTiO_3_ crystal has *d*_33_ = 83.7 pC N^–1^ and *d*_31_ = −27.5 pC N^–1^, whereas polyvinylidene difluoride films show *d*_33_ ≈ −20 pC N^–1^ and *d*_31_≈ 20 pC N^–1^ (refs. ^9,10^). These piezoelectric materials expand longitudinally along the electric field direction but contract transversally perpendicular to the field, or vice versa, exhibiting an electromechanical analogue of the positive Poisson’s ratio. Piezoelectric materials with longitudinal and transverse coefficients sharing the same sign, that is, both being positive or negative, have not yet been experimentally demonstrated, to the best of our knowledge. It has been theoretically predicted that some materials, such as orthorhombic HfO_2_ (belonging to the *Pca*2_1_ symmetry group) and quasi-two-dimensional ternary compounds, ASnX (A = Na, K; X = N, P; *P*6_3_*mc*) can possess both negative *d*_33_ and *d*_31_, though experimental evidence remains absent^11^. If realized, this exotic piezoelectric effect would be able to induce expansion or contraction in all directions under electric field stimulation. The effect would behave like the electrical analogue of auxetic materials showing a negative Poisson’s ratio^12,13^.

In addition to the bulk asymmetry discussed above, inversion symmetry breaking at interfaces can also induce non-centrosymmetry-related physical effects, including the piezoelectric effect^14–19^. Taking a Schottky junction as an example, the built-in field developed in the depletion region turns the semiconductor structure into a polar symmetry, regardless of the pristine symmetry of the semiconductor component. This gives rise to the interface piezoelectric effect, which arises from the synergy between the electrostriction effect and the electrical field, given as *d*_*ijk*_ = 2*M*_*jkil*_*E*_*l*_ (ref. ^14^) where *i*, *j*, *k* and *l* are direction index, described in more detail in the following. Concerning the case of the Schottky junction, its potential profile changes quadratically with distance from the metal–semiconductor interface, and the associated electric field decays linearly away from the interface. The effective electrical field is determined by the semiconductor properties, including the dielectric permittivity (*χ*) in the direction perpendicular to the interface (*χ*_3_), the dopant density (*N*_d_) and the built-in potential *V*_bi_. Thus, the dependence of the piezoelectric coefficient *d*_*ijk*_ of a Schottky junction on its semiconductor properties can be expressed as follows^14^:

1 $${d}_{{ijk}}={Q}_{{jki}3}{\chi }_{3}\sqrt{2q{N}_{{\mathrm{d}}}{\chi }_{3}{V}_{{{\mathrm{bi}}}}}$$

where *Q*_*jki*3_ is the electrostriction coefficient ($M_{ijkl}=Q_{ijkl}{\chi }_{k}{\chi }_{l}$) and *q* is the elementary charge. The subscripts *i*, *j*, *k* and *l* are the elements of the right-handed coordinate direction group {1, 2, 3}. The direction 3 always refers to the direction normal to the Schottky junction interface, while 1 and 2 refer to the in-plane directions. Clearly, the controlling factors of the interface piezoelectric effect are distinctive from those of the bulk counterpart. Since *χ*_3_ and $\sqrt{2q{N}_{{\mathrm{d}}}\,{\chi }_{3}{V}_{{{\mathrm{bi}}}}}$ are both positive, the sign of *d*_*ijk*_ is solely determined by the electrostriction coefficient *Q*_*jki*3_ (refs. ^20–22^). While the magnitude of the electrostriction coefficient can be optimized by chemical doping and the superlattice structure^23,24^, it is predicted that the sign of electrostriction can be tuned by crystallographic orientation^25,26^. We demonstrate that this crystallographic orientation dependence of the electrostriction effect can induce the auxetic piezoelectric effect at interfaces.

### Phenomenological theory of the auxetic piezoelectric effect

**Fig. 1:**
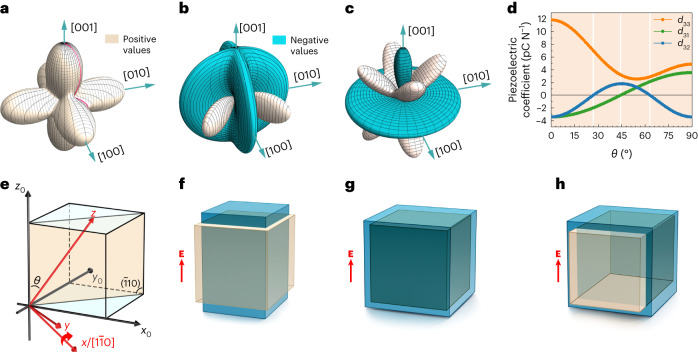
Phenomenological theory of the auxetic piezoelectric effect. **a**–**c**, Three-dimensional spherical polar plots of the crystallographic-orientation-dependent longitudinal piezoelectric coefficient *d*_33_ (**a**) and transverse coefficients *d*_31_ (**b**) and *d*_32_ (**c**) of Au/Nb:SrTiO_3_ Schottky junctions. The tan colour indicates positive values while cyan indicates negative values. **d**, Angle (*θ*)-dependent piezoelectric coefficients *d*_31_, *d*_32_ an*d d*_33_. **e**, Schematic showing the orientation varying from (001) to (110) within the $\left(\bar{1}10\right)$ plane with angle *θ* changing from 0° to 90°. Here, the *x* axis is along the $\left[1\bar{1}0\right]$ crystallographic orientation. **f**–**h**, Schematics showing the lattice deformation under an external electrical field (**E**) in the conventional piezoelectric effect (**f**), auxetic piezoelectric effect (**g**) and type II auxetic piezoelectric effect (**h**). The tan and cyan cubes respectively refer to the structures before and after applying the external electric field.

To resolve this crystallographic orientation dependence, we take Nb:SrTiO_3_ crystal as an example. Its electrostriction tensor ${Q}_{{ijkl}}^{0}$ in the (001) orientation has only three independent non-zero elements (that is, ${Q}_{11}^{0}$, ${Q}_{12}^{0}$ and ${Q}_{44}^{0}$) with its coordinate axes *x*_0_, *y*_0_ and *z*_0_ along the main crystallographic directions, that is, [100], [010] and [001]. When forming Schottky junctions on Nb:SrTiO_3_ crystal with an orientation different from the most usual (001) orientation, the electrostriction tensor ${Q}_{{ijkl}}^{{\prime} }$ of the newly oriented device with a coordinate set *xyz* will be different from ${Q}_{{ijkl}}^{0}$. The new coefficient ${Q}_{{ijkl}}^{{\prime} }$ can be derived from ${Q}_{{ijkl}}^{0}$ by tensor transformation using the correlation between their coordinate systems, which is given by

2 $${Q}_{{ijkl}}^{{\prime} }={R}_{{im}}{R}_{{jn}}{R}_{{kp}}{R}_{{lr}}{Q}_{{mnpr}}^{0}$$

where *R*_*ij*_ is the transformation matrix elements between coordinate sets *xyz* and *x*_0_ *y*_0_*z*_0_. Details are given in the Methods and Supplementary Fig. 1. The subscripts *m*, *n*, *p* and *r* also refer to {1, 2, 3} and the Einstein summation is used here. The crystallographic-orientation-dependent piezoelectric coefficients *d*_*ijk*_ can be obtained by putting equation (2) into equation (1) and using the usual parameters of the Au/Nb:SrTiO_3_ junctions prepared in our lab (for example, *χ*_3_ = 1.68 × 10^−9^ CV^−1^ m^−1^, *N*_d_ = 2.4 × 10^25^ m^−3^ and *V*_bi_ = 1.43 V). It is reasonable to assume that these parameters are independent of crystallographic orientation. As shown in the three-dimensional spherical polar plot (Fig. 1a), the longitudinal coefficient *d*_33_ of the Au/Nb:SrTiO_3_ junction retains a positive value in all the crystallographic orientations, with its maxima at the 〈001〉 directions. On the other hand, both the sign and magnitude of the transverse coefficients *d*_31_ and *d*_32_ vary with orientation (Fig. 1b,c). Interestingly, they turn from classical negative values to positive at certain orientations, with their positive maxima appearing at some 〈110〉 directions. To gain further insight, we extract a line profile of each piezoelectric coefficient, as indicated by the red line in Fig. 1a (line profiles are shown in Fig. 1d). Each profile corresponds to the crystal orientation (that is, the *z* axis) varying from (001) to (110) by sweeping angle *θ* between the *z* axis and *z*_0_ axis from 0° to 90° (Fig. 1e). The formula expressing the dependence of *d*_31_, *d*_32_ and *d*_33_ on the angle *θ* is given in the Methods.

As shown in Fig. 1d, *d*_33_ remains always positive with its maximum value at *θ* = 0° (that is, the (001) orientation) and minimum at *θ* ≈ 54.74° (corresponding to the (111) orientation). The *d*_31_ value turns from negative to positive at about 45°, whereas *d*_32_ turns positive at about 27° and then returns to negative at ≈ 63°. The *d*_31_ and *d*_32_ values are equal to each other at the (001) and (111) orientations due to the in-plane isotropy at these two orientations. Based on the signs of the coefficients, especially those of *d*_31_ and *d*_32_, one can classify the piezoelectric behaviour of the Au/Nb:SrTiO_3_ junction into three different categories. In the first category, *d*_33_ is positive while both *d*_31_ and *d*_32_ are negative, corresponding to *θ* in the range of 0–27°, which is the same as in the conventional piezoelectric effect (Fig. 1f). The second category corresponds to *θ* in the range of 45–63°, wherein *d*_31_, *d*_32_ and *d*_33_ are all positive. This means that an external field along the polar direction will induce expansion along all the directions, that is, the electrical analogue of a negative Poisson’s ratio (Fig. 1g). This is the auxetic piezoelectric effect as proposed. The third category shows the in-plane anisotropy with one transverse coefficient being positive while the other one remains negative, corresponding to *θ* in the ranges of 27–45° or 63–90°. This type of piezoelectric effect gives rise to an expansion along the longitudinal direction and one in-plane direction but contraction along the other transverse direction when an external electrical field is applied (Fig. 1h). This exotic effect is referred to here as the ‘type II’ auxetic piezoelectric effect. The role of the electrostriction tensor in the manifestation of the auxetic piezoelectric effect, especially the key features of the tensor elements that ensure this emerging phenomenon, is further discussed in the Methods. In short, it is the anisotropy of the electrostriction coefficients, especially the ratio between ${Q}_{11}^{0}$ and ${Q}_{44}^{0}$, that determines the manifestation of the auxetic piezoelectric effect. The larger the anisotropy, that is, the smaller ${Q}_{44}^{0}/{Q}_{11}^{0}$, the more likely a show of the auxetic piezoelectric effect. Note that the auxetic piezoelectric effect demonstrated here is totally absent in conventional piezoelectric bulk materials, such as BaTiO_3_, when one simply changes their device orientation, as is done here (Supplementary Fig. 2).

### Experimental demonstration of the auxetic piezoelectric effect

Guided by the above phenomenological theory, we chose Au/Nb:SrTiO_3_ Schottky junctions as the research object, with four crystallographic orientations: (001), (112), (111) and (110). These are representatives of the four angle ranges presented in Fig. 1d, which feature the piezoelectric effect with conventional, type II auxetic, auxetic and type II auxetic styles, respectively. These four interfaces with specific orientations possess a polar point symmetry of 4*mm*, *m*, 3*m* and *mm*2, respectively. The fabrication details of these junctions are given in the Methods. To demonstrate the manifestation of the auxetic piezoelectric effect, especially the sign of the coefficients, we first used the direct piezoelectric effect, which detects the short-circuit current induced by the application of a uniform dynamic force or stress. (The demonstration of the converse auxetic piezoelectric effect will be discussed later.) We specifically used an aluminium electrode as an ohmic contact with Nb:SrTiO_3_ crystals, playing the role of the counter electrode and forming a closed electrical circuit with the Schottky contact. The ohmic contact exhibits a negligible piezoelectric effect as demonstrated in our previous work^14^. To measure *d*_33_, we applied a dynamic force on the Schottky junction surface via two gold-coated ultra-flat sapphire wafers and simultaneously read the output current. For the transverse coefficients (*d*_31_, *d*_32_), the dynamic force was applied to two parallel side planes, and we monitored the current output by the Schottky junctions. Details of the set-up and measurement methods are given in the Methods and Supplementary Fig. 3. This approach, which characterizes the direct piezoelectric effect, is free from the entanglements of the electrostatic effect, electrochemical effect and strain-gradient effect, which usually occur in the characterization process of the converse piezoelectric effect^27,28^.

**Fig. 2:**
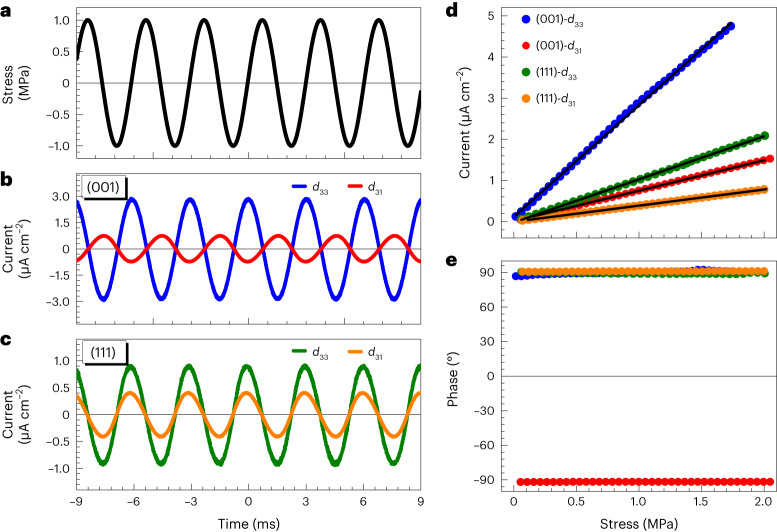
Experimental demonstration of the auxetic piezoelectric effect in Au/Nb:SrTiO_3_ Schottky junctions. **a**, Measured waveform of applied sinusoidal stress. The positive and negative signs refer to tensile and compressive stress, respectively. **b**, Current density waveform output by a (001)-oriented Au/Nb:SrTiO_3_ junction with the mechanical stress applied normal to the junction interface, that is, *d*_33_ (blue curve), and with the stress applied to the side planes of the sample, that is, *d*_31_ (red curve). **c**, Current density waveform output by a (111)-oriented Au/Nb:SrTiO_3_ junction in the *d*_33_ (green curve) and *d*_31_ (orange curve) measurement geometries, respectively. **d**,**e**, Load dependence of amplitude (**d**) and phase (**e**) of the current density output by the (001)- and (111)-oriented Au/Nb:SrTiO_3_ junctions.

We first compare the normal piezoelectric effect in a (001)-oriented Au/Nb:SrTiO_3_ junction with that of a (111)-oriented junction. Figure 2a,b shows both the longitudinal and transverse piezo-response of the (001)-oriented Au/Nb:SrTiO_3_ junction. For the sake of clarity, the amplitude of the dynamic stress applied to the samples is normalized to 1 MPa for both measurement geometries. The current induced in the longitudinal geometry (that is, *d*_33_) exhibits a 90° phase shift with respect to the dynamic stress, while current related to the transverse geometry (that is, *d*_31_) has a −90° phase shift, confirming the piezoelectric effect as the current generation mechanism and indicating the opposite signs between *d*_33_ and *d*_31_. The exact value of *d*_33_ and *d*_31_ can be obtained by measuring the output current density as a function of the applied stress. The amplitude of the current density generated by both *d*_33_ and *d*_31_ increases linearly with the amplitude of the applied stress, confirming once more the piezoelectric nature. Based on the relation *J*_3_ = *ωd*_3*i*_*σ*_*i*_, where *ω* = 2π*f* and *f* is the frequency of applied stress, *J*_3_ is the amplitude of the current density and *σ*_*i*_ is the stress amplitude, the value of the piezoelectric coefficients can be determined from the slope of *J*_*3*_ versus *σ*_*i*_. From the phase, which for both *d*_33_ and *d*_31_ remains constant over the measured stress range, we retrieve the sign of these coefficients. Thus, the values of *d*_33_ and *d*_31_ of the (001)-oriented Au/Nb:SrTiO_3_ junction are 13.1 pC N^–1^ and −3.4 pC N^–1^, respectively. The piezoelectric coefficients of this junction have been further confirmed by the frequency-dependent measurement, revealing a linear increase of the output current density with frequency at constant load (Supplementary Fig. 4). To compare these experimentally determined values with the theoretical values predicted by equations (1) and (2), the semiconductive properties of the junctions (that is, *χ*_3_, *V*_bi_ and *N*_d_) were determined via current–voltage characteristics, capacitance–voltage characteristics and the Hall effect (Supplementary Fig. 5). The theoretical piezoelectric coefficients yielded from equation (1) are *d*_33_ = 12.2 pm V^–1^ and *d*_31_ = −3.5 pm V^–1^, which are consistent with the experimental values. By contrast, the stress-induced current in the (111)-oriented Au/Nb:SrTiO_3_ junction shows a 90° phase shift in both the longitudinal and transverse measurement geometries with respect to the dynamic stress, indicating the same sign between *d*_33_ and *d*_31_ (Fig. 2c). This phase shift is almost constant over a wide stress range (Fig. 2e) and frequency range (Supplementary Fig. 4). The respective experimental values of the piezoelectric coefficients are *d*_33_ = 5 pC N^–1^ and *d*_31_ = 1.9 pC N^–1^ (Fig. 2d), which are also close to the predicted values of *d*_33_ = 6.0 pm V^–1^ and *d*_31_ = 2.9 pm V^–1^ (Supplementary Fig. 5 and Supplementary Table 2). This is a direct experimental proof of the auxetic piezoelectric effect, that is, both positive longitudinal and positive transverse coefficients, in the (111)-oriented Nb:SrTiO_3_ junction.

### Demonstration of the type II auxetic piezoelectric effect

**Fig. 3:**
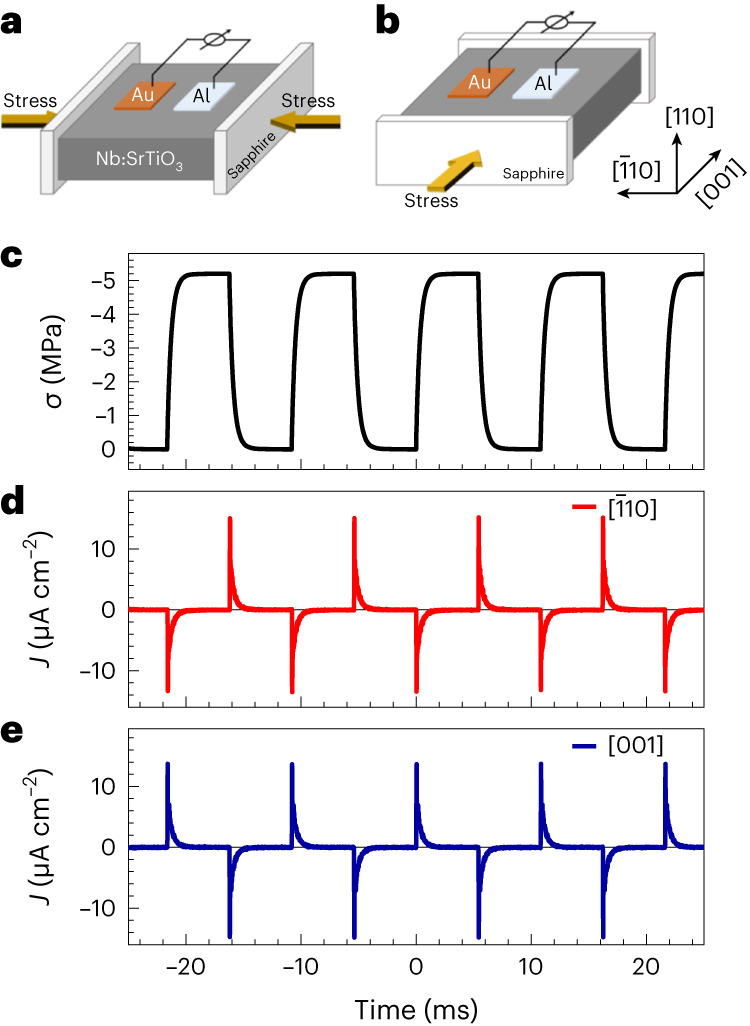
Demonstration of type ΙΙ auxetic piezoelectric effect in (110)-oriented Au/Nb:SrTiO_3_ Schottky junction. **a**,**b**, Schematics show the measurement geometries when force is applied along the [$1\bar{1}0$] in-plane direction to measure *d*_31_ (**a**) and [001] in-plane direction to measure *d*_32_ (**b**). **c**, Waveform of pulsed stress applied to side planes of the crystal. The stress amplitude (*σ*) has been normalized to 5 MPa. **d**,**e**, Piezoelectric current waveforms generated by the junction when stress is applied along the [$1\bar{1}0$] in-plane direction (**d**; signal proportional to *d*_31_) and [001] in-plane direction (**e**; signal proportional to *d*_32_).

We applied dynamic stress to the parallel side planes of the (110)-oriented Au/Nb:SrTiO_3_ junction along the [$1\bar{1}0$] and [001] crystallographic orientations, respectively, to characterize *d*_31_ and *d*_32_ (Fig. 3a,b). Figure 3c–e shows the transverse piezoelectric response under pulsed dynamic stress. When the pulsed stress is applied to the sample along the [$1\bar{1}0$] in-plane direction, the transient current output is negative; when the pulsed stress is withdrawn, the current output turns positive. However, when the stress is applied along the [001] direction, turning on the stress causes a positive peak current, while a negative peak current appears after turning off the stress. This result clearly confirms the opposite signs of the piezoelectric coefficients along the two orthogonal in-plane directions, that is, *d*_31_ and *d*_32_. The values of the measured piezoelectric coefficients under sinusoidal stress are *d*_31_ = 6.2 pC N^–1^, *d*_32_ = –6 pC N^−1^ and *d*_33_ = 9.3 pC N^–1^, which are also consistent with the values predicted by phenomenological theory, confirming the type II auxetic piezoelectric effect (Supplementary Figs. 6 and 7 and Supplementary Table 3). In addition, the (112)-oriented Au/Nb:SrTiO_3_ Schottky junction also shows the type II auxetic piezoelectric effect, as predicted. Details are given in Supplementary Fig. 8 and Supplementary Table 4.

### Key features of the auxetic piezoelectric effect

Having demonstrated the two types of auxetic piezoelectric effect in Nb:SrTiO_3_ Schottky junctions, we highlight four important features of the emergent phenomena. First, the auxetic piezoelectric effect demonstrated here also can be studied and elaborated on by density functional theory (DFT) calculations. Our DFT calculation replicates the auxetic behaviour of the (110)- and (111)-oriented SrTiO_3_ heterostructures (Supplementary Figs. 9 and 10). More importantly, our calculation indicates that the auxetic piezoelectric effect at the oxide interfaces mainly originates from the domination of the internal-strain relaxation mechanism over the clamped-ion contribution (Supplementary Table 5).

Second, given the inherent inversion symmetry breaking at the interface, the auxetic piezoelectric effect is expected to occur in various heterostructures consisting of diverse components regardless of their pristine symmetry. In this regard, we have discovered similar piezoelectric behaviours in (001)- and (100)-oriented Nb:TiO_2_ Schottky junctions. Rutile TiO_2_ has a centrosymmetric 4/*mmm* tetragonal structure, with a symmetry-forbidden piezoelectric effect. Once a Schottky junction is formed on its (001)-oriented surface, the interface symmetry reduces to the polar tetragonal 4*mm* point group. On the other hand, forming a Schottky junction on the (100)-oriented surface turns the interface symmetry to the orthorhombic *mm*2 point group, showing a piezoelectric effect with in-plane anisotropy. The piezoelectric coefficients of the (001)-oriented Au/Nb:TiO_2_ junction are measured to be *d*_31_ = *d*_32_ = 2 pC N^–1^ and *d*_33_ = 5.9 pC N^–1^, corresponding to the auxetic piezoelectric effect. The (100)-oriented Au/Nb:TiO_2_ junction has piezoelectric coefficients of *d*_31_ = 2.3 pC N^–1^, *d*_32_ = −1.5 pC N^–1^ and *d*_33_ = 3.4 pC N^–1^, that is, the type II auxetic piezoelectric effect (Supplementary Figs. 11 and 12). In addition to the built-in field in the Schottky junctions, interface asymmetry can also be induced by many other stimuli, such as a strain gradient^29^, compositional gradient^30^, tricolour superlattices^31^ and so on. Among them, the tricolour superlattices possess an inherent inversion asymmetry and electrical polarization due to the particular (ABCABC…)-style stacking of their three different components, offering a fertile platform to explore and exploit effects and functionalities induced by interface asymmetry^31^. In this regard, we have prepared and studied (001)- and (111)-oriented SrTiO_3_/Ba_0.6_Sr_0.4_TiO_3_/BaTiO_3_ superlattices with each layer of about 3 nm and a total thickness of about 100 nm. Details of the sample preparation and device characterization are given in the Methods. We discovered the auxetic piezoelectric effect in the (111)-oriented superlattice with effective piezoelectric coefficients of *d*_33_ = 6.6 pC N^–1^ and *d*_31_ = 3.3 pC N^–1^ (Supplementary Figs. 13–15). In comparison to Schottky junctions that have only one interface and limited functional thickness, the thickness of the tricolour superlattice can be designed as any rational value, which effectively turns the auxetic piezoelectric effect, which originates from interface asymmetry, to a bulk effect with no fundamental thickness limitation.

**Fig. 4:**
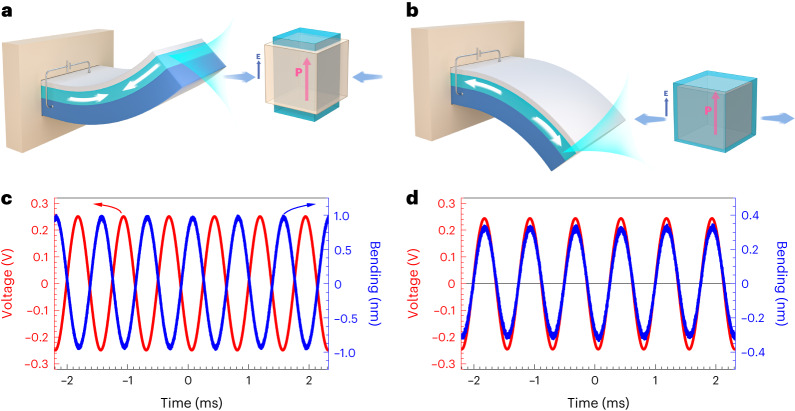
Demonstration of converse auxetic piezoelectric effect. **a**, Positive voltage induces upward bending in a cantilever with conventional piezo-active top layers. This cantilever is made of a piezo-inactive substrate, piezo-active layer and Pt top electrode. **b**, Auxetic piezoelectric effect induces downward bending. **P** refers to the induced electrical polarization arising from interface asymmetry. **c**, Waveform of applied a.c. voltage and induced bending displacement at the end of the cantilever made of a (001)-SrTiO_3_/Ba_0.6_Sr_0.4_TiO_3_/BaTiO_3_ superlattice grown on a Nb:SrTiO_3_ substrate. **d**, The a.c.-voltage-induced vibration of a (111)-oriented superlattice cantilever. The frequency of the excitation signal here is 1.33 kHz, and the amplitude is 0.25 V.

The auxetic piezoelectric effect demonstrated by the direct method also functions in the converse manner, converting electrical energy to mechanical energy. To demonstrate the converse auxetic piezoelectric effect, especially the transverse deformation under an external bias induced by *d*_31_, we developed a voltage-induced bending measurement using a cantilever device configuration, which is similar to that of a piezoelectric bimorph actuator (Fig. 4a,b). Due to the opposite signs of the transverse piezo-coefficients *d*_31_ (= *d*_32_), the conventional and auxetic piezo-effects will induce cantilevers that bend in opposite directions. Details of the measurement mechanism and technical details are given in the Methods. Figure 4c shows the vibration waveform of the (001)-oriented SrTiO_3_/Ba_0.6_Sr_0.4_TiO_3_/BaTiO_3_ superlattice under the application of an a.c. voltage. The a.c. voltage and induced dynamic bending are ∼180° out of phase. By contrast, the vibration of the (111)-oriented cantilever is fully in phase with the applied a.c. voltage (Fig. 4d). This difference relates to the opposite signs of the *d*_31_ values between the (001)- and (111)-oriented SrTiO_3_/Ba_0.6_Sr_0.4_TiO_3_/BaTiO_3_ superlattices and proves the auxetic nature of the converse piezoelectric effect in the (111)-oriented tricolour superlattice. Further characterization and discussion of the cantilever vibration measurements are given in the Methods.

**Fig. 5:**
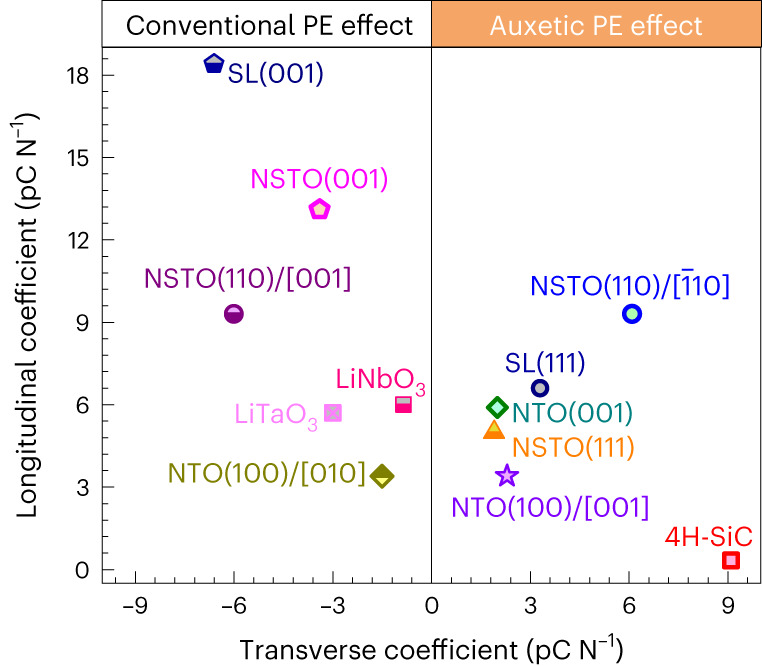
Summary of auxetic piezoelectric coefficients in heterostructures. Coefficients for conventional and auxetic piezoelectric (PE) effects are shown. NSTO refers to the Au/Nb:SrTiO_3_ Schottky junction; NTO refers to the Au/Nb:TiO_2_ Schottky junction; SL refers to the SrTiO_3_/Ba_0.6_Sr_0.4_TiO_3_/BaTiO_3_ tricolour superlattice; 4H-SiC refers to the Mo/4H-SiC junction. The index enclosed by parentheses refers to the device orientation, while the index enclosed in brackets refers to the transverse direction along which force is applied. Data of LiNbO_3_ and LiTaO_3_ crystals are from ref. ^32^.

The auxetic piezoelectric effect is of technical importance. For example, the exotic piezoelectric effect demonstrated here can be potentially applied in acoustic wave devices. The great flexibility in tuning its piezoelectric coefficients would facilitate the elimination of spurious modes of acoustic wave filters. As demonstrated in Fig. 1b,c and Supplementary Fig. 16, we can tune the transverse piezoelectric coefficients to zero in the 110)-Au/Nb:SrTiO_3_ junction if we set the in-plane edge directions along $\left[1\bar{1}\sqrt{2}\right]$ and $\left[\bar{1}1\sqrt{2}\right]$. In this specified crystallographic geometry design, an external field applied to the heterostructure will not induce transverse deformation, leaving this direction electromechanically inactive. In addition, being universal in nature, the auxetic piezoelectric effect allows us to explore and exploit this effect in materials compatible to complementary metal–oxide semiconductor technology. In this regard, we have explored heterostructures based on (0001)-oriented 4H-SiC. Despite being innately a polar material with 6*mm* point symmetry, the high carrier density and conductivity of the as-grown 4H-SiC prohibit its application in electromechanical devices. However, by forming a Schottky junction with Mo electrodes, we discovered the auxetic piezoelectric behaviour, with *d*_31_ = 9.1 pC N^–1^ and *d*_33_ = 0.32 pC N^–1^ (Fig. 5 and Supplementary Fig. 17). The fabrication details and generic characterization are given in the Methods. Interestingly, while the *d*_33_ value is rather small, the *d*_31_ value is very large. It is three times larger than that of LiTaO_3_ crystal and one order of magnitude larger than that of LiNbO_3_, the latter two being the workhorses in filters of communication devices^32^. Together with the wafer-fabrication-compatible nature and extraordinary mechanical properties, this auxetic piezoelectric effect of the Mo/SiC junction possesses great potential in applications such as all-semiconductor surface acoustic wave filters, sensors, actuators and so on^33^.

In summary, the pronounced tunability of the heterostructure interfaces facilitates the precise control of their electromechanical behaviors, leading to the discovery of the auxetic piezoelectric effect—a phenomenon that acts as the electrical counterpart to the negative Poisson’s ratio. These results will allow us to explore the piezoelectric effect in a wide range of established semiconductor materials and heterostructure configurations. Thus, the results offers innovative approaches to design and optimize electromechanical devices compatible with complementary metal–oxide semiconductor technology.

## Methods

### Phenomenological theory of auxetic piezoelectric effect

The transformation matrix **R** between the default coordinate set {*x*_0_, *y*_0_, *z*_0_} and the new set {*x*, *y*, *z*} used in Fig. 1 of the main text is given as follows:

3 $$\bf {R}=\left[\begin{array}{ccc}\frac{\cos \beta +\sin \beta }{\sqrt{2}} & \frac{-\cos \beta +\sin \beta }{\sqrt{2}} & 0\\ \frac{\cos \theta (\cos \beta -\sin \beta )}{\sqrt{2}} & \frac{\cos \theta (\cos \beta +\sin \beta )}{\sqrt{2}} & -\sin \theta \\ \frac{(\cos \beta -\sin \beta )\sin \theta }{\sqrt{2}} & \frac{(\cos \beta +\sin \beta )\sin \theta }{\sqrt{2}} & \cos \theta \end{array}\right]$$

where *θ* is the angle between the *z* axis and *z*_0_ axis, and *β* is the angle between *x* and the $[1\bar{1}0]$ crystallographic direction (Supplementary Fig. 1). With this setting, the *x* axis always remains in the *x*_0_*y*_0_ plane. Figure 1d in the main text corresponds to the scenario when *β* = 0 and the right-handed coordinate axis are rotated along the $[1\bar{1}0]$ axis.

Based on equations (1)–(3), the dependence of the Au/Nb:SrTiO_3_ Schottky junction piezoelectric coefficients *d*_333_, *d*_311_ and *d*_322_ on angle *θ* can be resolved as follows:

4 $$\begin{array}{l}{d}_{333}=\frac{1}{2}\left[2{Q}_{11}^{0}{\cos }^{4}\theta +\left({Q}_{11}^{0}+{Q}_{12}^{0}+2{Q}_{44}^{0}\right){\sin }^{4}\theta\right.\\\left. +\left({Q}_{12}^{0}+2{Q}_{44}^{0}\right){\sin }^{2}2\theta \right]{\chi }_{3}\sqrt{2q{N}_{{\mathrm{d}}}{\chi }_{3}{V}_{{{\mathrm{bi}}}}}\end{array}$$

5 $${d}_{311}=\left[{Q}_{12}^{0}{\cos }^{2}\theta +\frac{1}{2}\left({Q}_{11}^{0}+{Q}_{12}^{0}-2{Q}_{44}^{0}\right){\sin }^{2}\theta \right]{\chi }_{3}\sqrt{2q{N}_{{\mathrm{d}}}{\chi }_{3}{V}_{{{\mathrm{bi}}}}}$$

6 $$\begin{array}{l}{d}_{322}=\frac{1}{16}\left[3{Q}_{11}^{0}+13{Q}_{12}^{0}-6{Q}_{44}^{0}\right.\\\left.+\left(-3{Q}_{11}^{0}+3{Q}_{12}^{0}+6{Q}_{44}^{0}\right)\cos 4\theta \right]{\chi }_{3}\sqrt{2q{N}_{{\mathrm{d}}}{\chi }_{3}{V}_{{{\mathrm{bi}}}}}\end{array}$$

In addition to the centrosymmetric cubic semiconductor represented by Nb:SrTiO_3_, the phenomenological theory represented by equations (1) and (2) in the main text is applicable to semiconductors of any symmetry. In other words, the methodology developed here for Nb:SrTiO_3_ junctions as a generic example can be readily transferred to the case of other semiconductor Schottky junctions, such as the Nb:TiO_2_ junction, with minor modifications related to the dielectric permittivity. In the case of Nb:SrTiO_3_, its dielectric permittivity is independent of the crystallographic orientation and can be treated as a constant since its permittivity tensor has only one independent non-zero element. However, rutile TiO_2_ crystal has two independent non-zero elements in its permittivity tensor, that is, *χ*_11_ and *χ*_33_. Thus, the dielectric constant of TiO_2_ is dependent on the crystallographic orientation and cannot be treated as a simple scalar. One needs to calculate the orientation dependence of the dielectric permittivity via tensor transformation, as well, which is given by

7 $${\chi }_{{ij}}^{{\prime} }={R}_{{im}}{R}_{{jn}}{\chi }_{{mn}}$$

where *R*_*im*_ is the transformation matrix given in equation (3).

Note that the auxetic piezoelectric effect of a Schottky junction induces the same volume change as that of the conventional piezoelectric effect when an external voltage is applied. To elucidate the volume effect, we use Schottky junctions composed of a cubic semiconductor as the example, such as the Nb:SrTiO_3_ crystal. The dimension of the junctions stays constant as length × width × thickness = *a* × *a* × *t*. The volume variation when a unit external bias is applied to the junction along the thickness direction can be given as follows:

8 $$\Delta V={a}^{2}\left(1+{\frac{1}{t}d}_{31}\right)\left(1+{\frac{1}{t}d}_{32}\right)\left(t+{d}_{33}\right)-{a}^{2}t.$$

Since junction length/width and thickness are respectively on the scale of 100 μm and 100 nm, $1\gg {\frac{1}{t}d}_{31} \ {\mathrm{and}} \ 1\gg {\frac{1}{t}d}_{32}$. Thus, the above equation can be simplified to

9 $$\Delta V={a}^{2}({d}_{31}+{d}_{32}+{d}_{33}).$$

Note that, although the respective value of *d*_31_, *d*_32_ and *d*_33_ of the Schottky junctions changes while varying the crystallographic orientation as shown in Fig. 1 of the main text, the sum of *d*_31_, *d*_32_ and *d*_33_ retains a constant value, given as follows:

10 $${d}_{31}+{d}_{32}+{d}_{33}=({Q}_{11}^{0}+2{Q}_{12}^{0}){\chi }_{3}\sqrt{2q{N}_{{\mathrm{d}}}{\chi }_{3}{V}_{{{\mathrm{bi}}}}}.$$

This indicates that the auxetic piezoelectric effect induces the same volume change as the conventional piezoelectric effect in Schottky junctions consisting of the same type of cubic semiconductors. Therefore, the auxetic piezoelectric effect requires the same elastic energy cost compared to the conventional piezoelectric response in terms of volume change.

### The role of electrostriction coefficients on the auxetic piezoelectric effect

Variation of the electrostriction coefficients with respect to the crystallographic orientation plays an essential role in the manifestation of the auxetic piezoelectric effect. Thus, it is important to elucidate the way to specify or design materials with certain electrostriction tensors that can show the auxetic piezoelectric effect at their interfaces. To this end, we would like to first discuss the case of Schottky junctions composed of centrosymmetric cubic semiconductors, such as Nb:SrTiO_3_ and silicon. The methodology used for this type of material can then be generalized to other crystallographic structures, with some minor modifications.

The crystallographic-orientation-dependent piezoelectric coefficients *d*_33_, *d*_31_ and *d*_32_ are expressed by equations (4)–(6). Since ${\chi }_{3}\sqrt{2q{N}_{{\mathrm{d}}}{\chi }_{3}{V}_{{{\mathrm{bi}}}}}$ is always positive, the signs of *d*_33_, *d*_31_ and *d*_32_ are determined by the parts of these equations enclosed by brackets, that is, the transformed electrostriction coefficients. Here we set the ratio between the electrostriction coefficients as ${Q}_{12}^{0}/{Q}_{11}^{0}=\nu$ and ${Q}_{44}^{0}/{Q}_{11}^{0}=\eta$. Thus, the transformed electrostriction coefficients can be rewritten as follows:

11 $${Q}_{3333}^{{\prime} }=\frac{1}{16}{Q}_{11}^{0}\left[(9+7\nu +14\eta )+\left(1-\nu -2\eta \right)(4\cos 2\theta +3\cos 4\theta )\right]$$

12 $${Q}_{1133}^{{\prime} }=\frac{1}{4}{Q}_{11}^{0}\left[\left(1+3\nu -2\eta \right)-\left(1-\nu -2\eta \right)\cos 2\theta \right]$$

13 $${Q}_{2233}^{{\prime} }=\frac{1}{16}{Q}_{11}^{0}\left[\left(3+13\nu -6\eta \right)-3\left(1-\nu -2\eta \right)\cos 4\theta \right]$$

According to these equations, the possibility to have the auxetic piezoelectric effect is determined by the values of *ν* and *η*. The maximum and minimum values of ${Q}_{3333}^{{\prime} }$ by varying angle *θ* are ${Q}_{11}^{0}$ and $\frac{1}{16}(5.33+10.67\nu +21.34\eta ){Q}_{11}^{0}$, respectively. For most solid materials, the ratio *ν* between ${Q}_{12}^{0}$ and ${Q}_{11}^{0}$ can be approximated as Poisson’s ratio, which is usually in the range of −0.3 to −0.2. Also, ${Q}_{44}^{0}$ is usually of the same sign as ${Q}_{11}^{0}$, that is, *η* > 0. Thus, ${Q}_{3333}^{{\prime} }$ is always of the same sign as ${Q}_{11}^{0}$; namely, the bracketed part on the right side of equation (11) is always positive. Therefore, to have the type II auxetic piezoelectric effect, one needs to have

14 $$\left(1+3\nu -2\eta \right)-\left(1-\nu -2\eta \right)\cos 2\theta > 0,\theta \in [0,\uppi ]$$

or

15 $$\left(3+13\nu -6\eta \right)-3\left(1-\nu -2\eta \right)\cos 4\theta > 0,\theta \in [0,\uppi ].$$

To satisfy the above equations, one needs to, respectively, have the following:

16 $$\eta < \frac{1+\nu }{2}$$

or

17 $$\eta < \frac{3+5\nu }{6}.$$

Since *ν* is negative, (1 + *ν*)/2 is larger than (3 + 5*ν*)/6. Thus, the least condition to show the type II auxetic piezoelectric effect with one of *d*_31_ and *d*_32_ being positive is *η* < (1 + *ν*)/2. An example of the orientation-dependent electrostriction coefficient with *η* < (1 + *ν*)/2 is shown in Supplementary Fig. 18. In this case, ${Q}_{1133}^{{\prime} }$ turns positive, as ${Q}_{3333}^{{\prime} }$, when angle *θ* gets close to 90°, which gives rise to the type II auxetic piezoelectric effect. Once *η* decreases further, that is, (1 + 2*ν*)/2 < *η* < (3 + 5*ν*)/6, there will exist two angle regions that exhibit the type II auxetic piezoelectric effect, marked in grey in Supplementary Fig. 18c. The three-dimensional variation of the electrostriction coefficients corresponding to Supplementary Fig. 18 is given in Supplementary Fig. 19.

To have both *d*_31_ and *d*_32_ being the same sign as *d*_33_, *η* at least needs to satisfy the following:

18 $$\begin{array}{l}\left(1+3\nu -2\eta \right)-\left(1-\nu -2\eta \right)\cos 2\theta\\ =\left(3+13\nu -6\eta \right)-3\left(1-\nu -2\eta \right)\cos 4\theta > 0\end{array}$$

which results in

19 $$\cos 2\theta =-\frac{1}{3}$$

and

20 $$\eta < \frac{1+2\nu }{2}.$$

Supplementary Fig. 18d shows the variation of the electrostriction coefficients with respect to the angle *θ* when $\eta < \frac{1+2\nu }{2}$. The angle range to show the auxetic piezoelectric effect is marked by yellow, and the ranges to show the type II auxetic piezoelectric effect are marked by grey. Supplementary Fig. 19d is the corresponding three-dimensional version.

Overall, the manifestation of the auxetic piezoelectric effect in a Schottky junction made of semiconductors of cubic structure mainly depends on the relative value of ${Q}_{44}^{0}$ with respect to that of ${Q}_{11}^{0}$. The smaller ${Q}_{44}^{0}$, the easier to have an auxetic piezoelectric effect. To have *d*_33_, *d*_32_ and *d*_31_ of the same sign, one needs to have $\eta < \frac{1+2\nu }{2}$. Regarding semiconductors with other crystallographic structures, the same method exploited above can be used to identify the possibility of having an auxetic piezoelectric effect. One can use first-principles theory to calculate the electrostriction tensor of the material of interest and then use the tensor transformation method used in our work to decipher the crystallographic orientation dependence.

### Sample preparation

Nb:SrTiO_3_ and Nb:TiO_2_ single crystals with different orientations (SurfaceNet) were first cleaned by acetone, isopropyl and water in an ultrasonic bath. The (112)-oriented Nb:SrTiO_3_ substrates were prepared by Hefei Kejing Materials Technology. Afterward, the crystal surface was cleaned by oxygen plasma for 60 s before sputtering gold electrodes (Cressington sputter coater 208HR, or ET-NanoSputter from Anhui Epitaxy Technology). The ohmic contacts were formed by evaporating a Pt (40 nm)/Al (10 nm) bilayer on the crystal surface. The SrTiO_3_/Ba_0.6_Sr_0.4_TiO_3_/BaTiO_3_ superlattices were grown by the pulsed laser deposition method at 650 °C with an oxygen pressure of 0.15 mbar (ET-NanoPLD from Anhui Epitaxy Technology).

### Characterization methods of the auxetic piezoelectric effect

The piezoelectric coefficients were evaluated by a direct experimental approach, that is, by applying to the sample a homogeneous dynamic stress and measuring the short-circuit current as the main output. To measure *d*_33_, the Schottky junctions were clamped between two sapphire substrates coated with gold electrodes. Special care was taken to keep the sapphire substrate surface flat and clean as well as perfectly aligned and parallel to each other, to avoid any spurious effects arising from inhomogeneous strain. Au/Al_2_O_3_ plates applied the stress directly to the Schottky junction and collected the generated current (Supplementary Fig. 3a).

The measurement set-up for the transverse coefficients was the same as that used in our previous work (Supplementary Fig. 3b). The piezoelectric measurement set-up measures simultaneously both the piezoelectric current and the applied force (stress) using a force sensor. The generated current is first read by a transimpedance amplifier (DLPCA-200, Femto) and then displayed by oscilloscope as a waveform or measured by a lock-in amplifier as a vector signal.

### Demonstration of the converse auxetic piezoelectric effect

To detect the converse effect of the demonstrated auxetic piezoelectric effect, especially the transverse deformation under external bias induced by *d*_31_, we developed a voltage-induced bending measurement using a cantilever device configuration, which is similar to that of the piezoelectric bimorph actuator (Fig. 4a,b). We used a blade saw and polishing technique to prepare samples, prepared on Nb:SrTiO_3_ substrates into the cantilever geometry. The dimension of the (001)-oriented superlattice cantilever was about 3.5 mm × 1.2 mm × 0.15 mm, and that of the (111)-oriented one was about 4.0 mm × 1.6 mm × 0.12 mm.

Once a voltage parallel to the built-in polarization was applied to the piezo-active layer (that is, the superlattice or depletion layer of the Schottky junction) of the cantilever, the piezoelectric effect occurred and induced stress/strain. In the case of the (001)-oriented superlattice, this voltage induced an out-of-plane expansion and in-plane contraction, which generated an in-plane compressive stress to the top surface of the piezo-inactive substrate and bent the cantilever upward (Fig. 4a). By contrast, in the case of the (111)-oriented superlattice, due to the auxetic nature of its piezo-effect, the same applied voltage induced expansion in both the out-of-plane and in-plane directions, which exerted an in-plane tensile stress to the top layer of the cantilever and bent the cantilever downward (Fig. 4b). Thus, the (001)- and (111)-oriented cantilevers bent in opposite directions under the application of the same voltage, since the auxetic piezo-effect happened in the (111)-oriented samples. To demonstrate this scenario, we applied an a.c. voltage to both cantilevers and measured their vibration using an atomic force microscopy tip that contacted the end of the cantilevers. In our measurement set-up, a positive sign of the voltage represented its direction being parallel to the built-in polarization, that is, pointing from the bottom layer (that is, Nb:SrTiO_3_ here) to the top electrode (that is, Pt); a positive sign of the bending displacement at the end of the cantilevers meant a bending downward.

In addition to the vibration waveform shown in Fig. 4, we also measured the dependence of a cantilever vibration of both orientations on the a.c. voltage amplitude and frequency (Supplementary Fig. 20). The bending amplitude of both orientations increased linearly with the amplitude of the a.c. voltage. The vibration phase with respect to the a.c. voltage of the (001)-oriented cantilever almost retained a constant value of about 170°, while that of the (111)-oriented cantilever showed a phase of about −9°. This conforms to a negative sign of *d*_31_ in the (001)-oriented superlattice but a positive sign of *d*_31_ in the (111)-oriented superlattice, that is, the auxetic feature. Both cantilevers showed a resonant frequency at around 10 kHz. The most important feature in Supplementary Fig. 20c,d is the opposite change of the vibration phase between the two orientations. With increasing a.c. voltage frequency and passing through the resonance, the vibration of the (001) cantilever changed from out of phase to almost in phase, while that of the (111) cantilever evolved from in phase to out of phase, further conforming to the opposite signs of their transverse piezo-coefficients. Experimental data on the converse auxetic piezoelectric effect of Nb:SrTiO_3_ Schottky junctions are given in Supplementary Fig. 21.

Overall, these sophisticated characterizations clearly demonstrate the manifestation of both direct and converse auxetic piezoelectric effects in a symmetry-engineered tricolour superlattice, confirming the general nature of the auxetic piezoelectric effect. The results offer a promising platform to optimize the performance and exploit the potential of this emerging phenomenon.

## Online content

Any methods, additional references, Nature Portfolio reporting summaries, source data, extended data, supplementary information, acknowledgements, peer review information; details of author contributions and competing interests; and statements of data and code availability are available at 10.1038/s41563-023-01736-5.

### Supplementary information

Supplementary Information Supplementary Notes 1–14, Figs. 1–21 and Tables 1–5.

## Data Availability

The dataset that supports the findings of this study is available at the University of Warwick open access research repository (http://wrap.warwick.ac.uk/180001).

## References

[1] Curie J, Curie P (1880). Développement par compression de l'électricité polaire dans les cristaux hémièdres à faces inclinées. Bull. Minéral..

[2] Vanderbilt D (2000). Berry-phase theory of proper piezoelectric response. J. Phys. Chem. Solids.

[3] Saghi-Szabo G, Cohen RE, Krakauer H (1998). First-principles study of piezoelectricity in PbTiO_3_. Phys. Rev. Lett..

[4] Pan H (2019). Ultrahigh–energy density lead-free dielectric films via polymorphic nanodomain design. Science.

[5] Li F (2019). Giant piezoelectricity of Sm-doped Pb(Mg_1/3_Nb_2/3_)O_3_-PbTiO_3_ single crystals. Science.

[6] Katsouras I (2016). The negative piezoelectric effect of the ferroelectric polymer poly(vinylidene fluoride). Nat. Mater..

[7] You L (2019). Origin of giant negative piezoelectricity in a layered van der Waals ferroelectric. Sci. Adv..

[8] Fukada E, Yasuda I (1957). On the piezoelectric effect of bone. J. Phys. Soc. Jpn.

[9] Li Z, Grimsditch M, Xu X, Chan S-K (1993). The elastic, piezoelectric and dielectric constants of tetragonal PbTiO_3_ single crystals. Ferroelectrics.

[10] Jean-Mistral C, Basrour S, Chaillout J (2010). Comparison of electroactive polymers for energy scavenging applications. Smart Mater. Struct..

[11] Liu J, Liu S, Yang J-Y, Liu L (2020). Electric auxetic effect in piezoelectrics. Phys. Rev. Lett..

[12] Lakes R (1987). Foam structures with a negative Poisson’s ratio. Science.

[13] Evans KE, Alderson A (2000). Auxetic materials: functional materials and structures from lateral thinking!. Adv. Mater..

[14] Yang M-M, Luo Z-D, Mi Z, Zhao J, Alexe M (2020). Piezoelectric and pyroelectric effects induced by interface polar symmetry. Nature.

[15] Hwang HY (2012). Emergent phenomena at oxide interfaces. Nat. Mater..

[16] Soumyanarayanan A, Reyren N, Fert A, Panagopoulos C (2016). Emergent phenomena induced by spin–orbit coupling at surfaces and interfaces. Nature.

[17] Hellman F (2017). Interface-induced phenomena in magnetism. Rev. Mod. Phys..

[18] Akamatsu T (2021). A van der Waals interface that creates in-plane polarization and a spontaneous photovoltaic effect. Science.

[19] Ma C (2022). Intelligent infrared sensing enabled by tunable moiré quantum geometry. Nature.

[20] Devonshire A (1954). Theory of ferroelectrics. Adv. Phys..

[21] Uchino K, Nomura S, Cross LE, Jang S, Newnham R (1980). Electrostrictive effect in lead magnesium niobate single crystals. J. Appl. Phys..

[22] Li F, Jin L, Xu Z, Zhang S (2014). Electrostrictive effect in ferroelectrics: an alternative approach to improve piezoelectricity. Appl. Phys. Rev..

[23] Zhang H (2022). Atomically engineered interfaces yield extraordinary electrostriction. Nature.

[24] Korobko R (2012). Giant electrostriction in Gd-doped ceria. Adv. Mater..

[25] Kvasov A, Tagantsev AK (2012). Positive effective *Q*_12_ electrostrictive coefficient in perovskites. J. Appl. Phys..

[26] Nye, J. F. *Physical Properties of Crystals: Their Representation by Tensors and Matrices* (Oxford Univ. Press, 1985).

[27] Seol D, Kim B, Kim Y (2017). Non-piezoelectric effects in piezoresponse force microscopy. Curr. Appl. Phys..

[28] Abdollahi A, Domingo N, Arias I, Catalan G (2019). Converse flexoelectricity yields large piezoresponse force microscopy signals in non-piezoelectric materials. Nat. Commun..

[29] Lee D (2011). Giant flexoelectric effect in ferroelectric epitaxial thin films. Phys. Rev. Lett..

[30] Damodaran AR (2017). Large polarization gradients and temperature-stable responses in compositionally-graded ferroelectrics. Nat. Commun..

[31] Sai N, Meyer B, Vanderbilt D (2000). Compositional inversion symmetry breaking in ferroelectric perovskites. Phys. Rev. Lett..

[32] Smith R, Welsh F (1971). Temperature dependence of the elastic, piezoelectric, and dielectric constants of lithium tantalate and lithium niobate. J. Appl. Phys..

[33] Matthews, H. *Surface Wave Filters: Design, Construction, and Use* (John Wiley & Sons, 1977).

