# Supplementary material for: Auxetic piezoelectric effect in heterostructures
Source: Nat Mater. 2023 Nov 30;23(1):95–100. doi: 10.1038/s41563-023-01736-5 (PMC10769876; doi:10.1038/s41563-023-01736-5)
Supplement: Supplementary file 1 — Supplementary Notes 1–14, Figs. 1–21 and Tables 1–5. [file 41563_2023_1736_MOESM1_ESM.pdf]

nature materials

Article https://doi.org/10.1038/s41563-023-01736-5

Auxetic piezoelectric effect in heterostructures

In the format provided by the authors and unedited

Table of Contents

Table S1. Summary of measured longitudinal and transverse piezoelectric coefficients......2

Note S1: Crystallographic orientation resolved interface piezoelectric coefficients.............3

Note S2: Crystallographic orientation dependent piezo-coefficients in bulk materials.........4

Note S3: Characterization methods of the auxetic piezoelectric effect..................................6

Note S4: Frequency dependent piezoelectric response..7

Note S5: Electrical characterization of the Au/Nb:$SrTiO_3$ Schottky junctions 8

Note S6: Piezoelectric characterization of the (110)-oriented Au/Nb:$SrTiO_3$ junction.......10

Note S7: Characterization of the (112)-oriented Au/Nb:$SrTiO_3$ junction...........................12

Note S8: Density functional theory calculations of auxetic piezoelectric effect 14

Note S9: Piezoelectric effect of rutile Au/Nb:TiO_2 junctions...18

Note S10: Observation of auxetic piezoelectric effect in tricolour superlattice 19

Note S11. In-plane orientation dependent piezoelectric coefficients...................................22

Note S12: Preparation and characterization of Mo/4H-SiC junctions 24

Note S13: The role of electrostriction coefficients on the auxetic piezoelectric effect.........25

Note S14: Converse auxetic piezoelectric effect 27

References 29

Table S1. Summary of measured longitudinal and transverse piezoelectric coefficients

Substance	d_{31} [pC/N]	d_{33} [pC/N]	References
$BaTiO_3$	-78	190	*(S1)*
$Ba_2NaNb_5O_{15}$	-7	37	*(S2)*
$Ba_{0.5}Ca_{0.5}TiO_3$	-58.3	204	*(S3)*
$BaTeMo_2O_9$	-1.8	0.3	*(S4)*
$Cs_2S_2O_6$	-0.3	13.7	*(S5)*
$KNbO_3$	-31	51	*(S6)*
$LiNbO_3$	-1	6	*(S7)*
$LiTaO_3$	-2	8	*(S7)*
$Li_2B_4O_7$	-1.84	8.76	*(S8)*
Li_2GeO_3	-4.0	11.1	*(S9)*
Li_2SiO_3	-2.0	7.6	*(S10)*
Na_2GeO_3	-5.8	11.7	*(S10)*
$PbTiO_3$	-27.5	83.7	*(S11)*
$PbMg_{1/3}Nb_{2/3}O_3$	-72	240	*(S12)*
$PbNb_2O_6$	-9.5	85	*(S13)*
PZT-4	-123	289	*(S13)*
PIN-PMN-PT	-650	1300	*(S14)*
PIN-PSN-PT	-365	770	*(S15)*
PMN-PT	-393	855	*(S16)*
PZNT91/09	-1700	2500	*(S17)*
PMN-BT-PT	-170	530	*(S18)*
$YCa_4O(BO_3)_3$	-0.77	1.4	*(S19)*
$(Ba_{0.85}Ca_{0.15})(Zr_{0.1}Ti_{0.9})O_3$	-182	510	*(S20)*
$(Ca_{0.28}Ba_{0.72})_{0.25}(Sr_{0.6}Ba_{0.4})_{0.75}Nb_2O_6$	-31.1	94.3	*(S21)*
$(K_{0.5}Na_{0.5})(Nb_{0.97}Sb_{0.03})O_3$	-82	208	*(S22)*
$0.92Pb(Zn_{1/3}Nb_{2/3})O_3$-$0.08PbTiO_3$	-1455	2890	*(S23)*
$0.38Bi(Ga_{0.125}Sc_{0.875})O_3$-$0.62PbTiO_3$	-142	420	*(S24)*
BeO	-0.12	0.24	*(S25)*
ZnO	-5.0	12.4	*(S26)*
AgI	-9.1	19.1	*(S27)*
AlN	-2.65	5.53	*(S28)*
AlScN	-4	9.9	*(S29)*
$Y_{0.375}Al_{0.625}N$	-8.65	17.5	*(S30)*
CdS	-5.18	10.32	*(S31)*
CdSe	-3.92	7.84	*(S32)*
GaN	-1.9	3.7	*(S33)*
PVDF	20	-20	*(S34)*
P(VDF/TrFE) (75/25)	7	-38	*(S35)*
PVDF-HFP	30	-24	*(S36)*

Note S1: Crystallographic orientation resolved interface piezoelectric coefficients

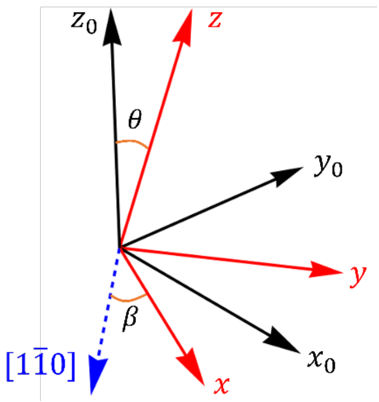

Fig.S1. **Schematic** showing the geometrical correlation between new coordinate set $\{x\ y\ z\}$ and default set $\{x_0, y_0, z_0\}$.

Note S2: Crystallographic orientation dependent piezo-coefficients in bulk materials

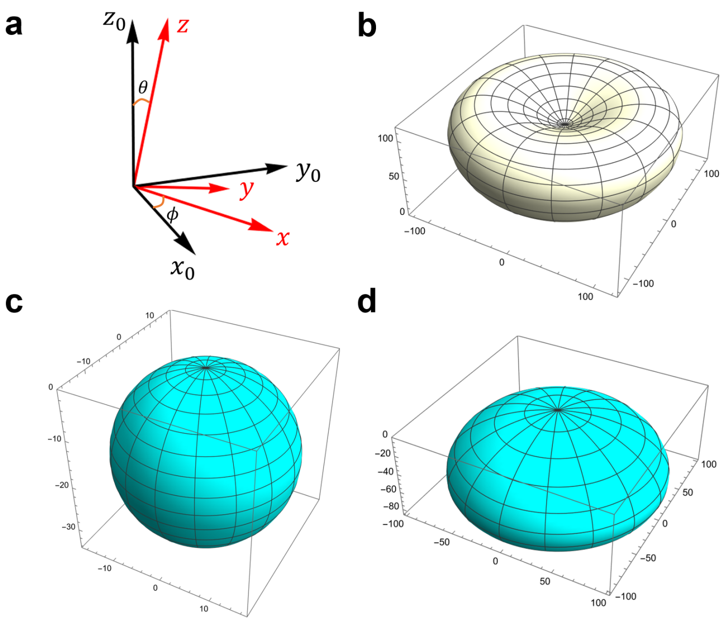

Fig. S2. Crystallographic orientation dependent piezoelectric coefficients of $BaTiO_3$ crystal. a), Schematic showing the geometrical correlation between new coordinate set $\{x\ y\ z\}$ and default set $\{x_0, y_0, z_0\}$. In this rotation configuration, x-axis retains in the $(x_0 y_0)$ plane. ϕ refers to the angle between x-axis and x_0-axis. θ refers to the angle between z-axis and z_0-axis. 3D-dimensional spherical polar plot of **b**) d_{33}, **c**) d_{31}, and **d**) d_{32}. Here angle θ is set in the range of $(0, \pi/2)$. The light-yellow indicates positive value while cyan refers to negative value of piezoelectric coefficients.

Manipulating crystallographic orientation has also been utilized to optimize piezoelectric coefficients and electromechanical coupling factor in conventional piezoelectric materials with bulk inversion asymmetry, such as quartz (*S37*) and $BaTiO_3$ (*S38*). Here we take tetragonal $BaTiO_3$ crystal as an example and show that its longitudinal piezoelectric coefficient d_{33} and transverse coefficients d_{31} & d_{32} are always of opposite signs measured at any cutting orientation directions. The piezoelectric tensor of tetragonal $BaTiO_3$ in its conventional coordinate axis is of the form:

$$d_{ij} = \begin{bmatrix} 0 & 0 & 0 & 0 & d_{15} & 0 \\ 0 & 0 & 0 & d_{15} & 0 & 0 \\ d_{31} & d_{31} & d_{33} & 0 & 0 & 0 \end{bmatrix} \qquad \text{S1}$$

where $d_{15} = 392\ pC/N$, $d_{31} = -34.5\ pC/N$, $d_{33} = 85.6\ pC/N$. (*S39*) To calculate the cutting orientation dependent piezoelectric coefficients of $BaTiO_3$ crystal, we use here a transformation matrix R_{ij} given as:

$$R_{ij} = \begin{bmatrix} cos\phi & -sin\phi & 0 \\ cos\theta sin\phi & cos\theta cos\phi & -sin\theta \\ sin\theta sin\phi & cos\phi sin\theta & cos\theta \end{bmatrix} \quad \text{S2}$$

The definition of θ and ϕ is shown in Fig. S2a. The coordinate set is first rotated along z_0 -axis by ϕ and then rotated along the x-axis by θ.

Therefore, the cutting orientation dependent piezoelectric coefficient $d'_{ijk}(\theta, \phi)$ can be calculated by following equation:

$$d'_{ijk} = R_{im}R_{jn}R_{kp}d_{mnp} \quad \text{S3}$$

We found that

$$d_{33} = cos\theta(221.55 - 135.95cos\theta^2 + 135.95sin\theta^2) \quad \text{S4}$$

$$d_{31} = -34.5cos\theta \quad \text{S5}$$

$$d_{32} = cos\theta(-170.45 + 135.95cos\theta^2 - 135.95sin\theta^2) \quad \text{S6}$$

These piezoelectric coefficients are independent of angle ϕ and their 3-dimensional spherical polar plots are shown in Fig.S2 b-d. Clearly, d_{33} is always of opposite sign with respect to d_{31} & d_{32}.

Note S3: Characterization methods of the auxetic piezoelectric effect

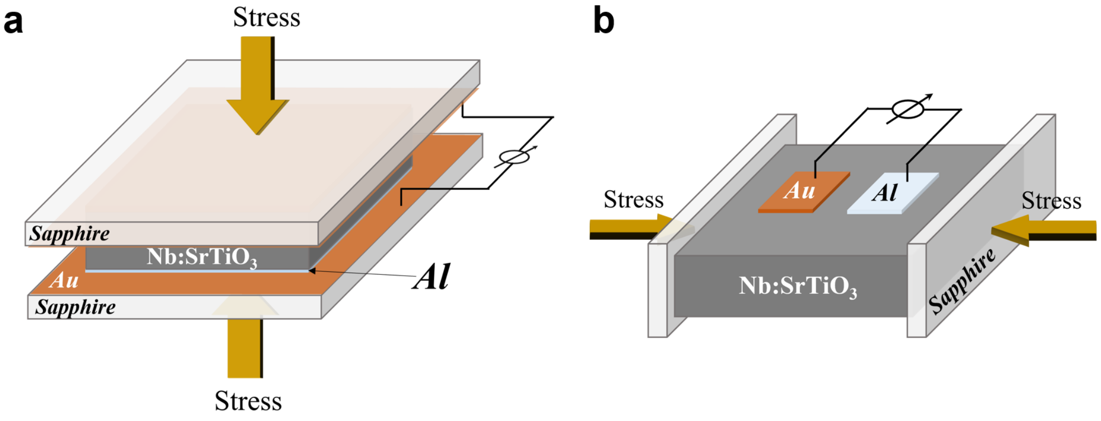

Fig. S3. Schematics showing the measurement geometry to characterize piezoelectric coefficients. **a**), d_{33} measurement setup and **b**), d_{31} measurement setup.

Note S4: Frequency dependent piezoelectric response

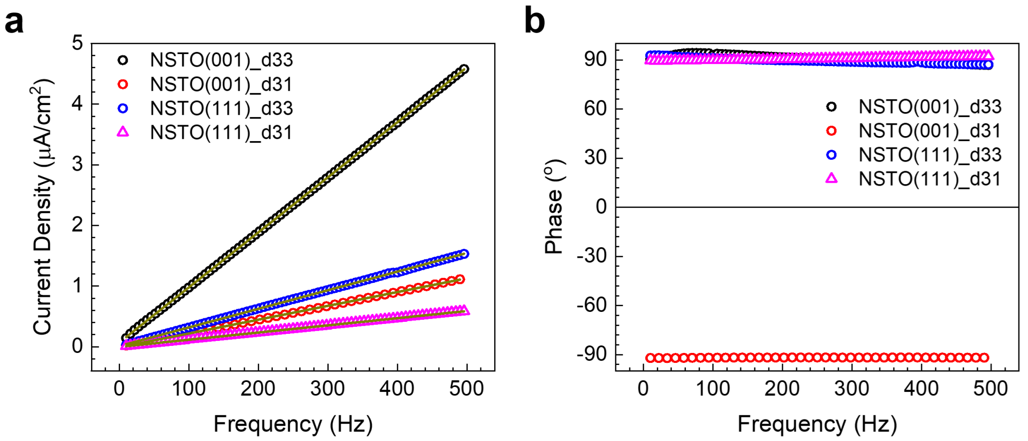

Fig. S4. Frequency dependent piezoelectric response of (001)- and (111)-oriented Au/Nb:SrTiO$_3$ junctions. The stress for all measurements is set as 1 MPa. The solid curves in **A** are linear fits.

As shown in Fig. S4a, the current induced by the interface piezoelectric effect in both (001)- and (111)-oriented Au/Nb:SrTiO$_3$ junctions increases linearly while increasing stress frequency. This is consistent with the equation: (*S40*)

$$J_3 = 2\pi f d_{3i}\sigma_i \qquad \text{S7}$$

where J_3 is the amplitude of current density output by Schottky junctions, f is the frequency of the stress and σ_i is the amplitude of the stress. At all frequencies the phase of piezo-current generated by d_{33} of (001)-oriented Au/Nb:SrTiO$_3$ and both d_{33} and d_{31} of (111)-oriented Au/Nb:SrTiO$_3$ stays at ~90°, while that of d_{31} in (001)-oriented remains constant of $-90°$. This is consistent with that shown in Fig. 2 of the main text.

Note S5: Electrical characterization of the Au/Nb:SrTiO3 Schottky junctions

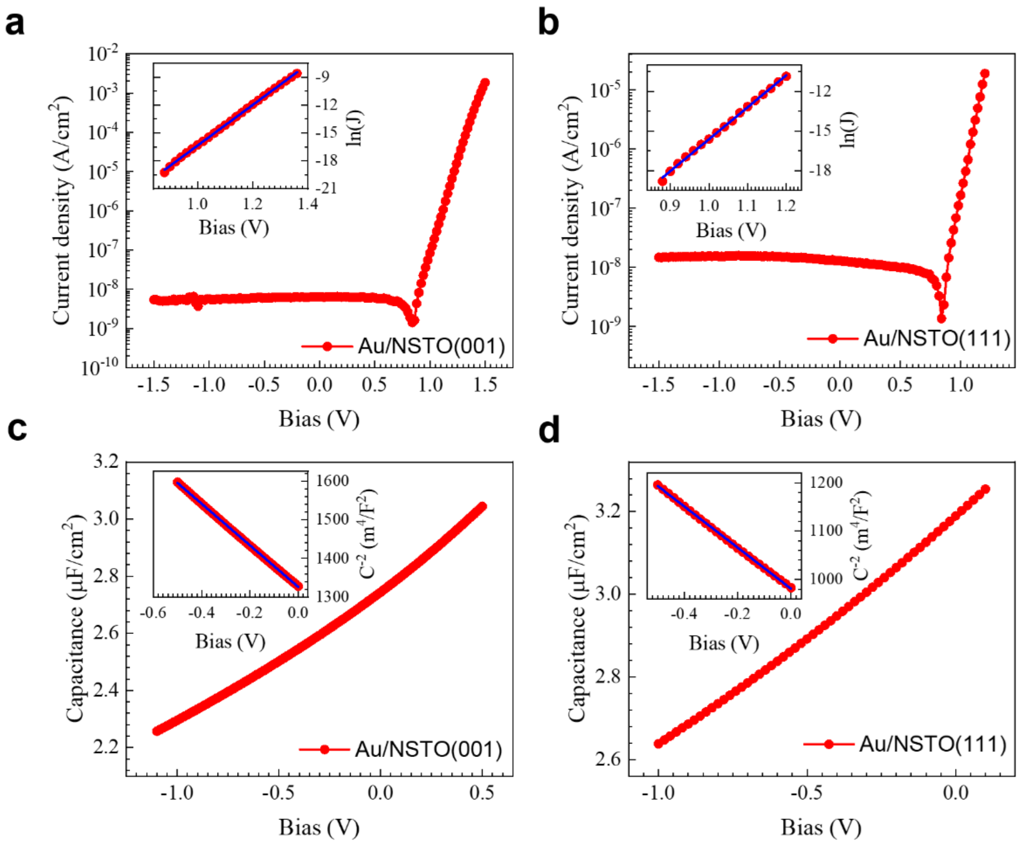

Fig. S5. Electrical characterization of the Au/Nb:SrTiO3 Schottky junctions. Current-voltage current of **a**), (001)-Au/Nb:SrTiO3 and **b**), (111)-Au/Nb:SrTiO3. The inset is the ln(J) vs applied bias in the forward bias regime and its linear fit. The capacitance vs bias curves of **c**), (001)-Au/Nb:SrTiO3 and **d**), (111)-Au/Nb:SrTiO3. The inset is the C^{-2} vs applied voltage plot in the reverse bias regime and the linear fit.

The physical properties of the Schottky junctions can be characterized by classical semiconductor measurements. The dielectric permittivity $\chi_3$and built-in potential V_{bi} can be determined from the current-voltage (J vs V) and capacitance-voltage (C vs V) curves based on following equations: (*S41*)

$$J = A^{*}T^{2}\exp\left(-\frac{q\Phi_B}{k_B T}\right)\left[\exp\left(\frac{qV}{nk_B T}\right) - 1\right] \qquad \text{S8}$$

$$\frac{1}{C^2} = \frac{2n^2 V_{bi}}{qN_d\varepsilon_r\varepsilon_0} - \frac{2n}{qN_d\varepsilon_r\varepsilon_0}V \qquad \text{S9}$$

where J is the current density, A^* is the Richardson constant, T is temperature, k_B is the Boltzmann constant, Φ_B is the potential barrier height, n is the ideality factor, C is the capacitance per unit area and ε_r is the relative dielectric permittivity. The ideality factor n can be acquired by fitting the $\ln J\ \ vs\ V$ as shown in the insets of Fig. S5a, B. Given the values of N_d and n, the effective permittivity χ_n can be obtained by $C^{-2}\ vs\ V$ fitting as shown in the insets of Fig. S5c, d.

The dopant density N_d can be approximated as the effective carrier density in Nb:$SrTiO_3$ measured by the Hall effect. From standard Hall measurements at room temperature with magnetic field up to 2 T, the carrier density of (001)- and (111)-oriented Nb:$SrTiO_3$ crystals are respectively $2.16 \times 10^{25} m^{-3}$ and $2.43 \times 10^{25} m^{-3}$. Based on these parameters, the values of d_{31} and d_{33} of (001)- and (111)-oriented junction can be predicted based on Eq. (1) of the main text. All these values are given in Table S2.

Table S2. Values of physical parameters of (001)- and (111)-oriented Au/Nb:$SrTiO_3$ junctions

	n	N_d ($\times 10^{25} m^{-3}$)	ε_r	V_{bi} (V)	d_{31}(pm/V)	d_{33} (pm/V)
(001)	1.83	2.16	221	1.37	-3.5	12.2
(111)	1.64	2.43	226	1.49	2.9	6.0

Note S6: Piezoelectric characterization of the (110)-oriented Au/Nb:SrTiO₃ junction

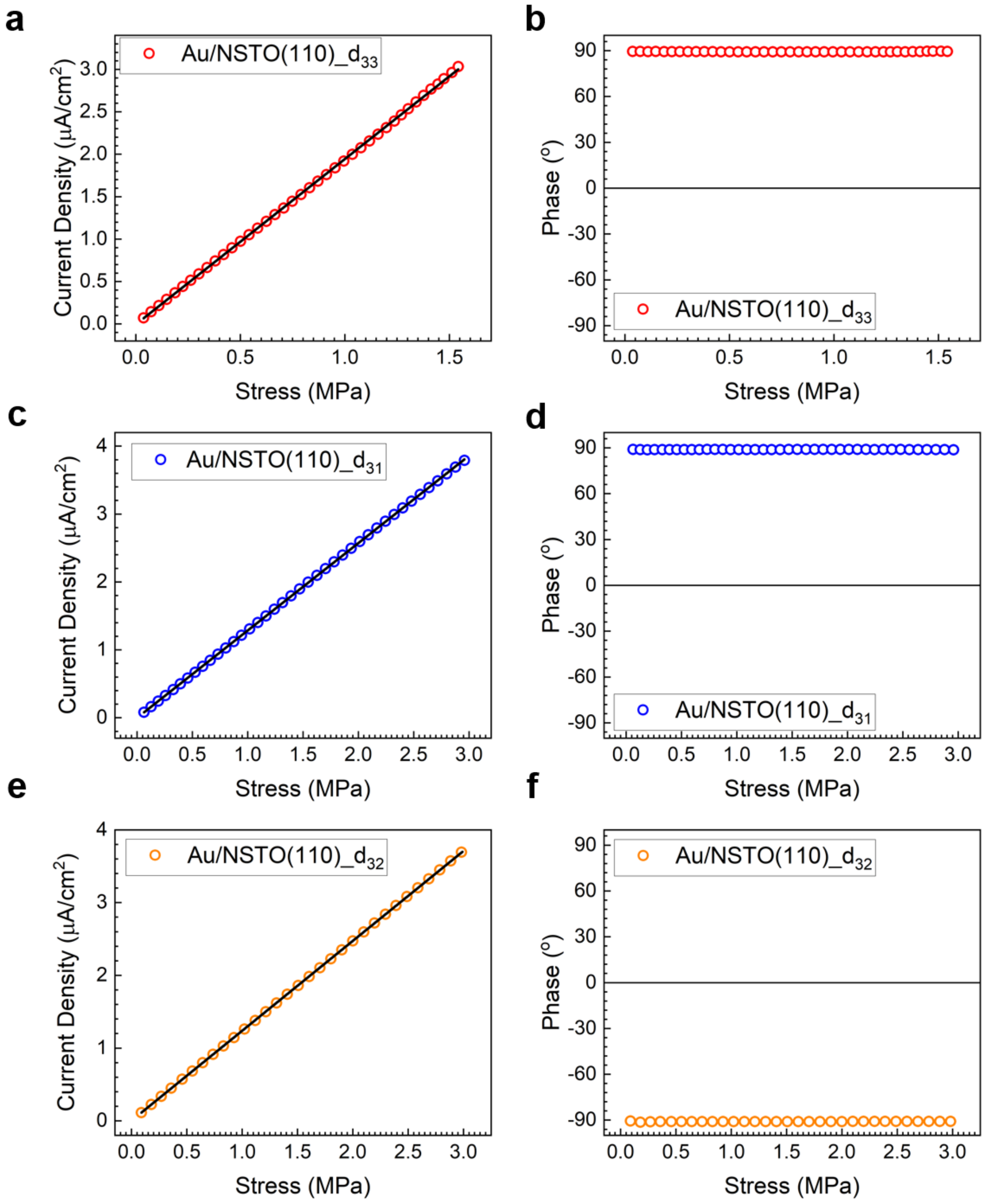

Fig. S6. Piezoelectric characterization of (110)-oriented Au/Nb:SrTiO₃ junction. Stress dependent amplitude of current density induced by **a**) d_{33}, **c**) d_{31} and **e**) d_{32}. The phase dependence on stress is shown respectively in **b**), **d**) and **f**).

The values of the longitudinal and transverse piezoelectric coefficients, i.e., d_{31}, d_{32} and d_{33}, of (110)-oriented Au/Nb:$SrTiO_3$ junction are quantitatively characterized by using sinusoidal stress wave (see Fig. S6). The stress-induced current increases linearly with the

amplitude of the applied stress, confirming their piezoelectric nature. The phase of the piezoelectric current of both d_{31} and d_{33} is 90° with respect to the dynamic stress, indicating its positive sign of their values, while the current phase of d_{32} is about $-90°$, indicating its negative sign. Based on these measurements, the values of d_{31}, d_{32} and d_{33} are 6.2 pC/N, -6 pC/N and 9.3 pC/N, respectively.

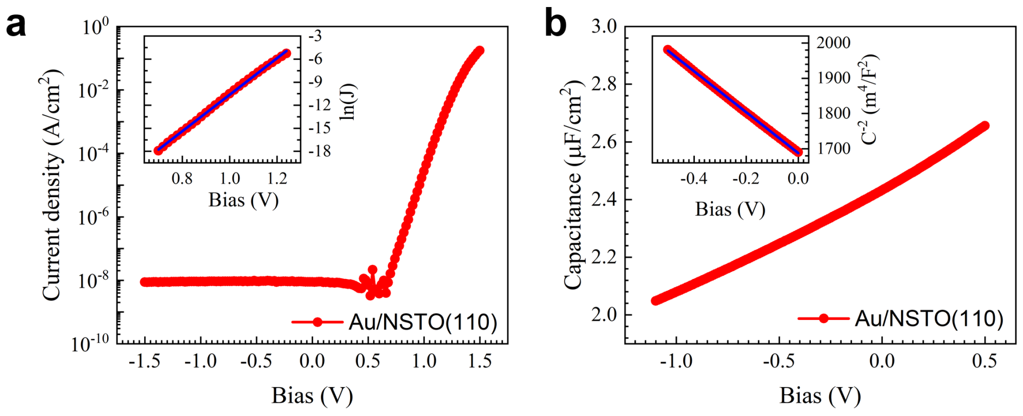

Fig. S7. Electrical characterization of (110)-oriented Au/Nb:$SrTiO_3$ junction. **a**), Current-voltage curves. Inset is the ln(J) vs voltage curve and its linear fit. **b**), The capacitance-voltage curve. Inset is the C^{-2} vs voltage curve and its linear fit.

The electrical properties of (110)-oriented Au/Nb:$SrTiO_3$ have also been characterized by current-voltage curve and capacitance-voltage curve (see Fig. S7). The fitted values of its parameters are shown in Table S3.

Table S3. Values of physical parameters of (110)-oriented Au/Nb:$SrTiO_3$ junctions

	n	N_d ($\times 10^{25} m^{-3}$)	ε_r	V_{bi} (V)	d_{31} (pm/V)	d_{32} (pm/V)	d_{33} (pm/V)
(110)	1.64	1.34	330	1.36	5.2	-5.2	7.1

Note S7: Characterization of the (112)-oriented Au/Nb:$SrTiO_3$ junction

We have customized (112)-oriented Nb:$SrTiO_3$ single crystal substrates from Hefei Kejing Matl. Tech. Co., Ltd. that corresponds to the angle $\theta \cong 35.3°$. The effective dopant density of these new substrates is measured as $1.26 \times 10^{25}\ m^{-3}$. Its Schottky junctions with platinum electrodes are characterized by current-voltage curve and capacitance-voltage curve as shown in **Fig. S8** a, b. Based on these measurements, the electrical parameters of these (112)-oriented Au/Nb:STO junctions derived using Eq. S8 and S9 in the Supplementary Information are summarized in Table S4. With these parameters, its piezoelectric coefficients can be predicted using Equation 1 of the main text, which are given as: $d_{33} = 6.3\ pC/N$, $d_{31} = -1.4\ pC/N$, $d_{32} = 1.5\, pC/N$. The experimental characterization of the piezoelectric effect of the (112)-orientations are shown in Fig. S8 c-f. The piezoelectric coefficients are measured as $d_{33} = 4.2\, pC/N$, $d_{31} = -1.0\ pC/N$, $d_{32} = 0.93\, pC/N$, which are fairly close to the predicted values. Thus, the experimental results are consistent with our phenomenological theory that further consolidates our work.

Table S4. Values of physical parameters of (112)-oriented Au/Nb:$SrTiO_3$ junctions

n	N_d ($\times 10^{25} m^{-3}$)	ε_r	V_{bi} (V)	d_{31} (pm/V)	d_{32}(pm/V)	d_{33} (pm/V)
1.46	1.26	328	1.14	-1.4	1.5	6.3

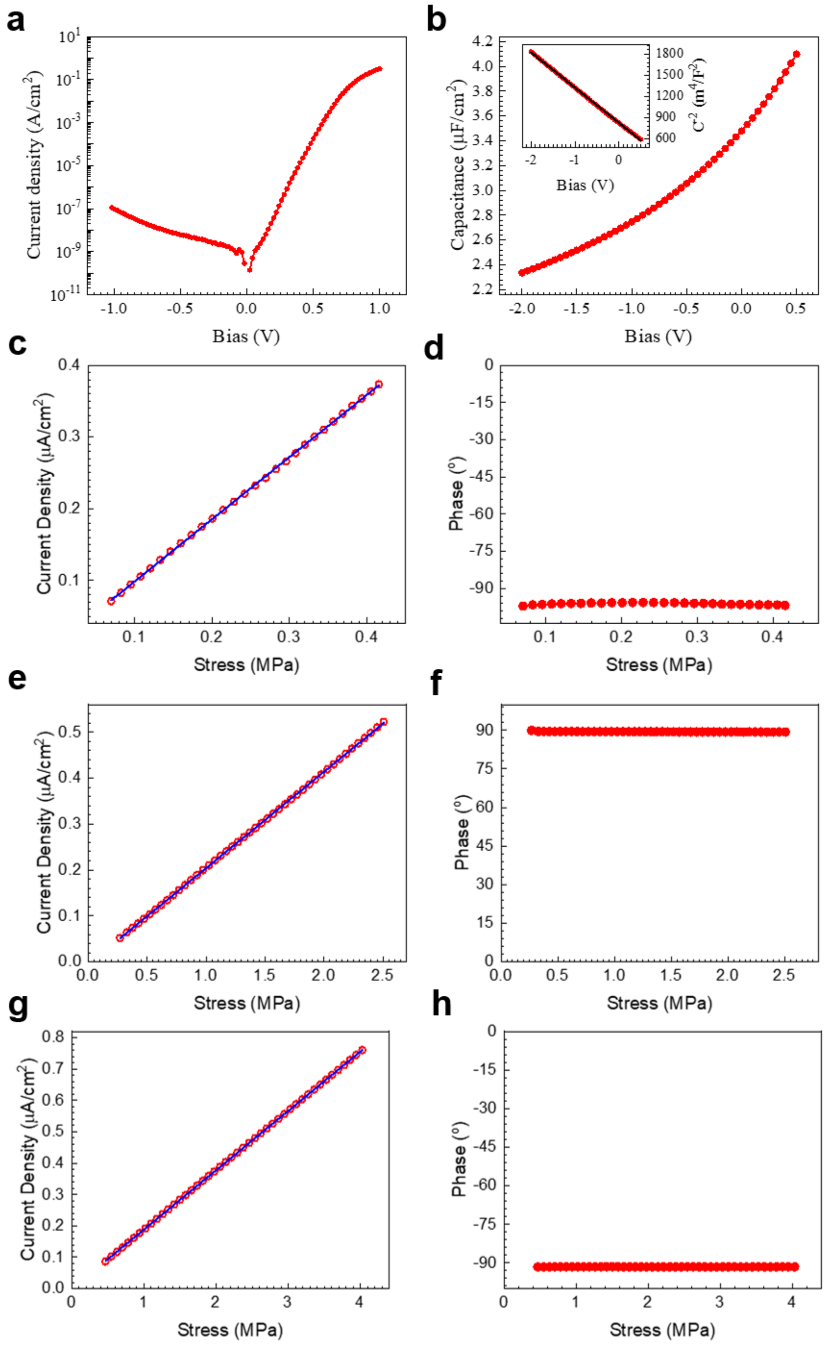

Fig.S8. Electrical and piezoelectric characterization of (112)-oriented Au/Nb:SrTiO$_3$ junction. **a**, Current vs voltage characterization and **b**, capacitance vs voltage curve of the junction. Stress dependent amplitude of current density induced by **c**) d_{33}, **e**) d_{31} and **g**) d_{32}. The phase dependence on stress is shown respectively in **d**), **f**) and **h**).

Note S8: Density functional theory calculations of auxetic piezoelectric effect

Density functional theory (DFT) calculations were carried out using the Vienna *ab initio* simulation package (VASP) with the projector-augmented wave (PAW) method (*S42, S43*). The Perdew-Burke-Ernzerhof exchange-correlation functional for solid (PBEsol) (*S44*) has been employed in this calculation. The electronic wave-functions are expanded in a plane-wave basis set with a cut-off energy of 600 eV. The optimized lattice parameters of bulk $SrTiO_3$ and Au are 3.896 Å and 4.083 Å, respectively, implying a relatively small lattice mismatch (4.58%) between these two materials. Thus, a good epitaxial relationship can be kept by stacking a $SrTiO_3$ slab and an Au slab with the same orientations, i.e., Au/$SrTiO_3$ (001), Au/$SrTiO_3$ (110) and Au/$SrTiO_3$ (111). The Brillouin zones of the (001), (110) and (111) interface structures are sampled with Γ-centred (6×6×1), (6×5×1) and (5×3×1) k-point meshes, respectively. All the interface structures are fully relaxed until the atomic forces are converged to 0.01 eV/Å.

The $SrTiO_3$ (001) slab is stacked by alternating charge neutral layers of SrO and TiO_2, while the (110) slab consists of alternating charged $SrTiO^{4+}$ and O_2^{4-} layers, and the (111) slab consists of alternating charged Ti^{4+} and SrO_3^{4-} layers (*S45*). To avoid the introduction of an internal electric field arising from asymmetric surface terminations, we construct each heterostructure by connecting a symmetric $SrTiO_3$ slab and an Au slab (with the same terminations on both sides). The $SrTiO^{4+}$ termination in (110) slab and the Ti^{4+} termination in (111) slab naturally exhibits a *n*-type-like feature and leads to a charge transfer from the positively charged terminations of $SrTiO_3$ to the metal electrode, which is a typical character of a Schottky contact, as confirmed by our calculations. In contrast, the negatively charged terminations O_2^{4-} and SrO_3^{4-} exhibits a *p*-type-like feature with the charge transfer direction from the metal electrode to the negatively charged terminations. It is worthy to note that as both SrO and TiO_2 terminations in (001) slab is charge neutral, the charge transfer between these terminations and the metal electrode is very small. In order to model the Schottky contact between STO and Au observed in experiments, we focus on the interface structures that have the correct charge-transfer direction (from semiconductor to metal electrode) and large charge-transfer magnitude, i.e., $SrTiO^{4+}$-terminated Au/$SrTiO_3$(110) and Ti^{4+}-terminated Au/$SrTiO_3$ (111) interfaces. As demonstrated in the main text, these two orientations give rise to the auxetic piezoelectric effect.

According to the modern theory of polarization (*S46, S47*), only the change of polarization is well defined and can be associated with the experimentally measurable quantity, i.e.,

switching current. Hence, we calculate the polarization as the interface charge transfer per area given by following equation:

$$P_3 = \frac{\Delta Q}{A} \quad \text{S10}$$

where ΔQ is the interface charge transfer which can be obtained by employing the Bader charge analysis (*S12*), and A is the interface area. Interface piezoelectricity can be described by the piezoelectric strain coefficient, which is defined as the ratio of interface polarization P_3 with respect to an applied uniaxial stress σ_i, i.e.,

$$d_{3i} = \frac{\partial P_3}{\partial \sigma_i} \quad \text{S11}$$

or by the piezoelectric stress coefficient, which is defined as the ratio of interface polarization P_3 with respect to an applied uniaxial strain ε_i, i.e.,

$$e_{3i} = \frac{\partial P_3}{\partial \epsilon_i} \quad \text{S12}$$

The sign and magnitude of the piezoelectric stress coefficient are determined by two types of contributions, i.e., "clamped-ion" ($e_{3i}^{(0)}$) and "internal-strain" ($e_{3i}^{(i)}$) contributions, which can be resolved by the DFT calculation (S48).

The atomic structures of the $SrTiO^{4+}$-terminated Au/$SrTiO_3$ (110) interface are shown in Fig. S9a. Fig. S9b shows the dependence of its interface polarization on uniaxial stress (upper panel, d_{3i}), on uniaxial strain with ions relaxed (middle panel, e_{3i}) and with ions clamped (lower panel, $e_{3i}^{(0)}$). Meanwhile, the calculation results of the (111)-oriented Au/$SrTiO_3$ interface with Ti^{4+}-termination are given in Fig. S10. The interface polarization P_3 exhibits good linear relationship with the uniaxial stress and uniaxial strain applied along all the directions. The piezoelectric strain coefficient d_{3i} and piezoelectric stress coefficient e_{3i} can respectively be estimated by performing linear fitting between the polarization P_3 and the uniaxial stress σ_i and uniaxial strain ϵ_i (see Table S5). It is remarkable that the calculated piezoelectric strain coefficients are of the same signs and same order of magnitudes with both experimental and phenomenological results.

The piezoelectric stress coefficients of the $SrTiO^{4+}$-terminated Au/$SrTiO_3$(110) interface are calculated to be $e_{31} = 22.06\ \mu\mathrm{C/cm^2}$, $e_{32} = -20.80\ \mu\mathrm{C/cm^2}$ and $e_{33} = 52.34\ \mu\mathrm{C/cm^2}$. Calculation yields piezoelectric stress coefficients $e_{31} = 28.90\ \mu\mathrm{C/cm^2}$, $e_{32} = 20.79\ \mu\mathrm{C/cm^2}$ and $e_{33} = 30.51\ \mu\mathrm{C/cm^2}$ for Ti^{4+}-terminated Au/$SrTiO_3$(111) interface. Interestingly, these values have the same signs with the piezoelectric strain coefficients and

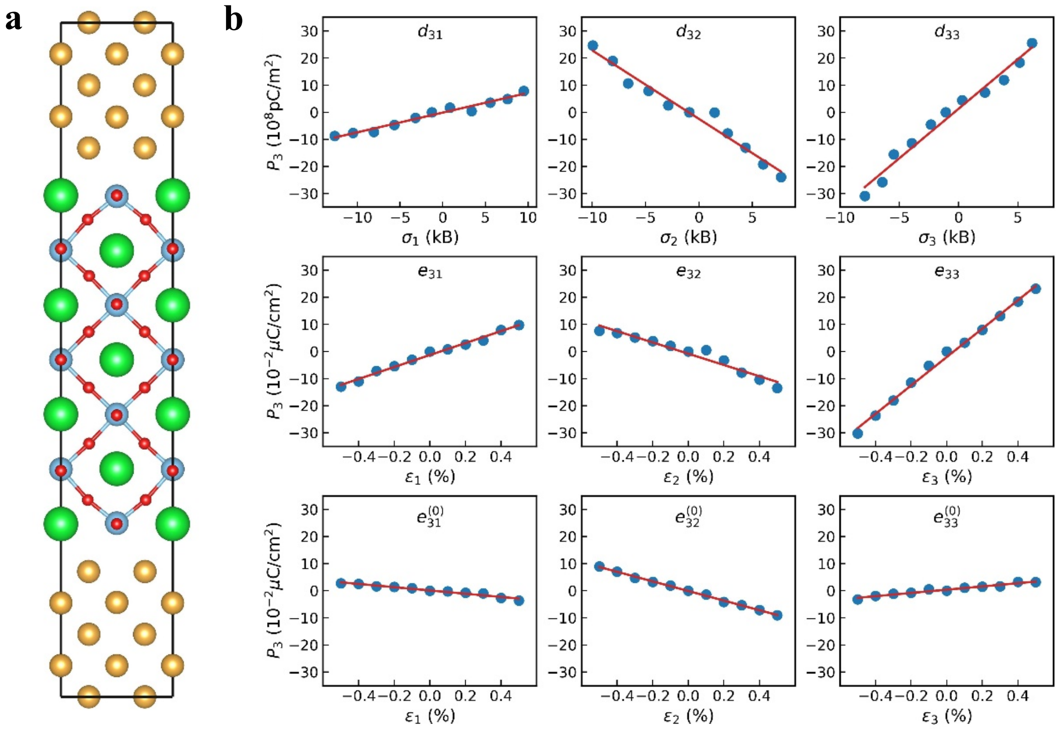

Fig. S9. DFT calculation results of (110)-oriented Au/SrTiO$_3$ interface. **a**), Schematic illustrates the atomic structure of the heterostructure. **b**), Dependence of interface polarization on uniaxial stress (upper panel), on uniaxial strain with ions relaxed (middle panel) and with ions clamped (lower panel). The Sr, Ti, O, and Au atoms are depicted as green, light blue, red, and golden spheres, respectively. The polarization of unstrained interface is set to zero.

more importantly, their relative magnitudes are more consistent with experimental results as $e_{31} \approx -e_{32}$ for (110) interface and $e_{31} \approx e_{32}$ for (111) interface. We propose that in our heterostructure models, due to the existence of metal layer which has better malleability than semiconductor layer, the application of uniaxial stress unavoidably introduces a relatively large strain in the other two lattice directions. It implies that in comparison to uniaxial stress condition, the uniaxial strain condition (with the lattice fixed) is closer to the experimental reality. Hence, the calculated piezoelectric stress coefficients have a better consistency with experimental results than the piezoelectric strain coefficients. Furthermore, the piezoelectric stress coefficients can be decomposed into the clamped-ion and internal-strain contributions. we find that all the positive interface piezoelectricity, i.e., e_{31} and e_{33} of (110) interface, e_{31}, e_{32} and e_{33} of (111) interface, arises from the domination of positive internal-strain term. In contrast, the clamped-ion term only dominates the negative e_{32} of (110) interface.

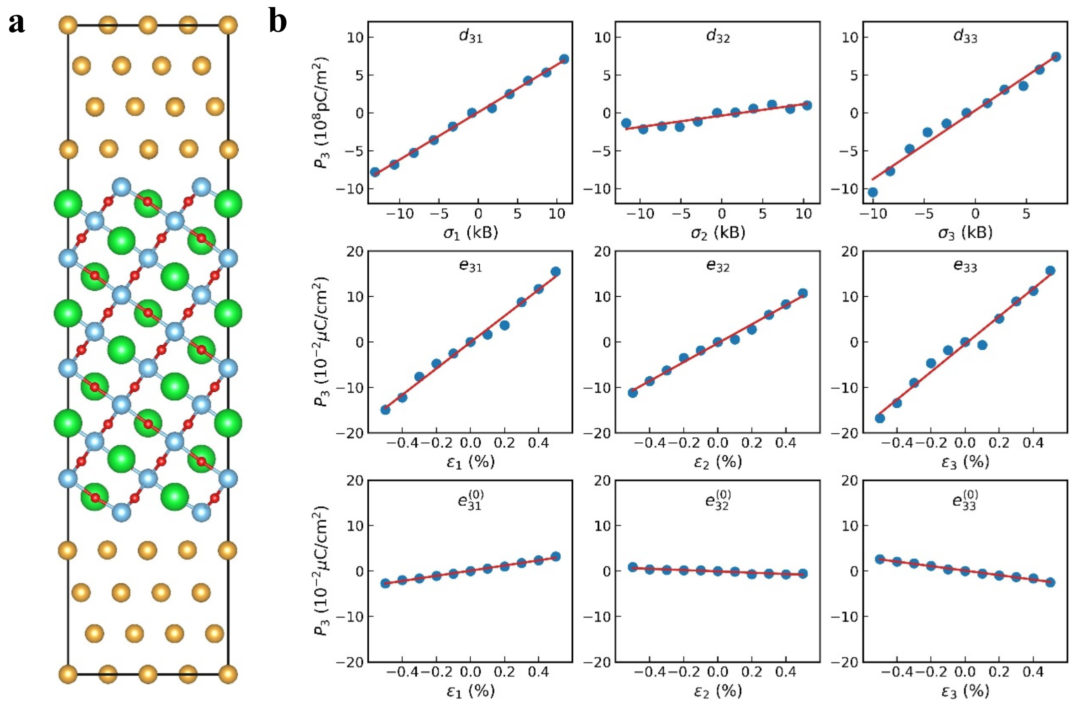

Fig. S10. DFT calculation results of (111)-oriented Au/$SrTiO_3$ interface. **a**), Schematic illustrates the atomic structure of the heterostructure. **b**), Dependence of interface polarization on uniaxial stress (upper panel), on uniaxial strain with ions relaxed (middle panel) and with ions clamped (lower panel). The Sr, Ti, O, and Au atoms are depicted as green, light blue, red, and golden spheres, respectively. The polarization of unstrained interface is set to zero.

Table S5. Calculated piezoelectric strain coefficients d_{3i} (in units of pC/N) and piezoelectric stress coefficients e_{3i} (in units of μC/cm²). The clamped-ion" ($e_{3i}^{(0)}$) and "internal-strain" ($e_{3i}^{(i)}$) contributions of $SrTiO^{4+}$-terminated Au/ STO(110) and Ti^{4+}-terminated Au/STO (111) interfaces have also been resolved.

**Orientation**	**(110)**			**(111)**		
d_{3i}	d_{31}	d_{32}	d_{33}	d_{31}	d_{32}	d_{33}
	0.73	−2.53	3.65	0.63	0.15	0.91
e_{3i}	e_{31}	e_{32}	e_{33}	e_{31}	e_{32}	e_{33}
Total	22.06	−20.80	52.34	28.90	20.79	30.51
Clamped-ion	−6.00	−17.71	5.99	5.67	−1.44	−4.95
Internal-strain	28.06	−3.09	46.35	23.23	22.23	35.46

Note S9: Piezoelectric effect of rutile Au/Nb:TiO_2 junctions

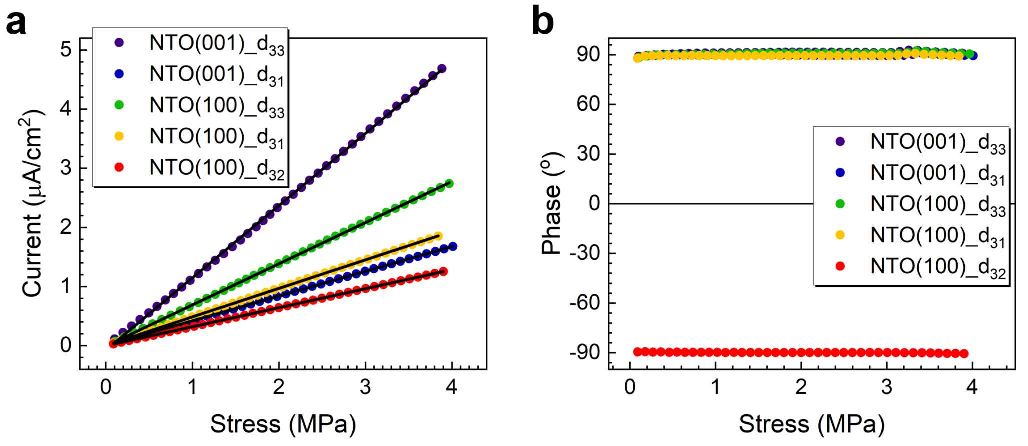

Fig. S11. Piezoelectric characterization of the Au/Nb:TiO_2 junctions. **a**), Dependence of induced current amplitude on the amplitude of applied stress. The black lines are linear fits. **b**), Phase of piezoelectric current with respect the applied sinusoidal stress wave at all applied stress values.

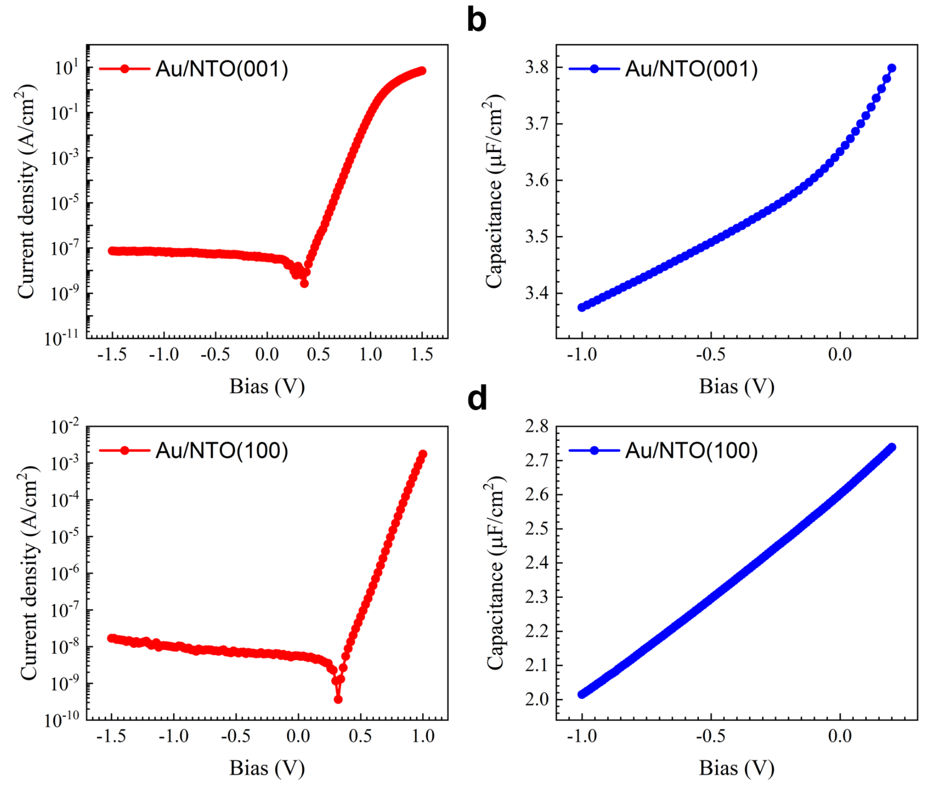

Fig. S12. **Electrical characterization of the Au/Nb:TiO_2 junctions**. **a**), **c**) Current-voltage curve and **b**), **d**) capacitance-voltage curve. **a**) and **b**) are acquired on (001)-oriented Au/Nb:TiO_2 Schottky Junction. **c**) and **d**) are for (100)-oriented Au/Nb:TiO_2 Schottky Junction.

Note S10: Observation of auxetic piezoelectric effect in tricolour superlattice

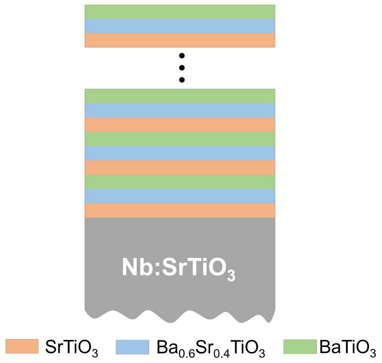

Fig. S13: Schematic showing the [$SrTiO_3$/$Ba_{0.6}Sr_{0.4}TiO_3$/$BaTiO_3$] tricolour superlattice grown on Nb:$SrTiO_3$ substrates.

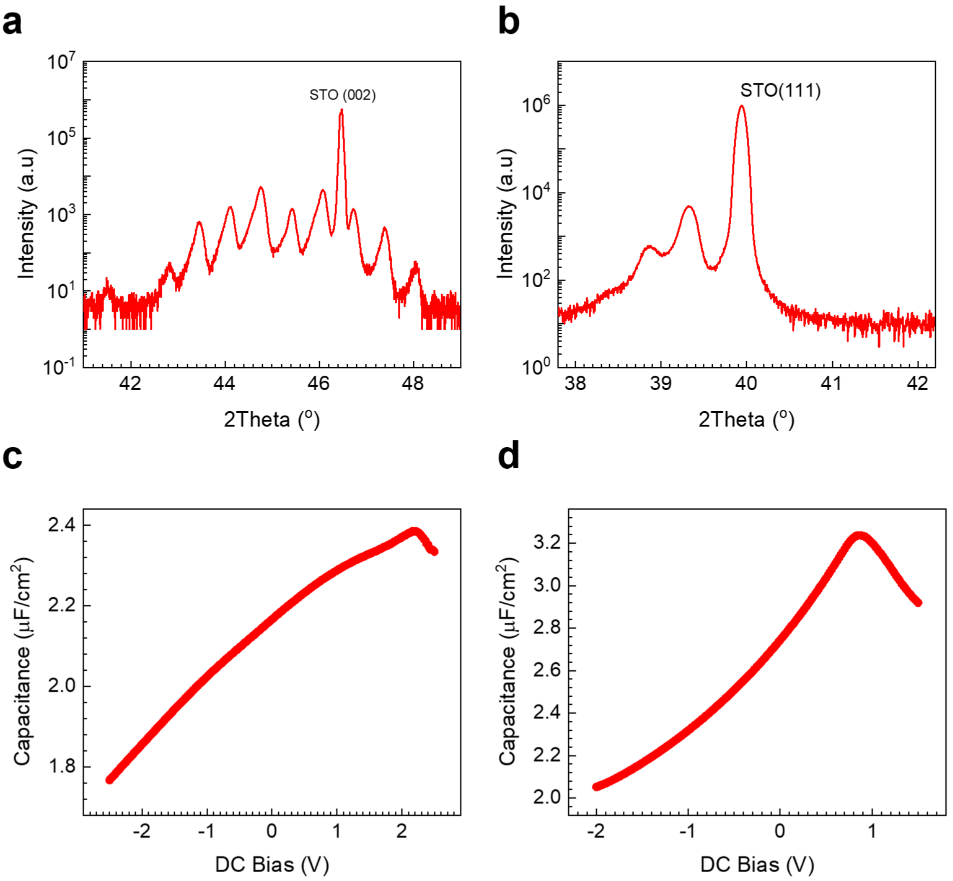

Fig. S14. Structure and capacitance characterization of [$SrTiO_3$/$Ba_{0.6}Sr_{0.4}TiO_3$/ $BaTiO_3$]$_{10}$ superlattices. $2\theta - \omega$ XRD scan of **a)**, (001) and **b)**, (111)-oriented superlattices. The capacitance vs external DC bias characterization of **c)**, (001) and **d)**, (111)-oriented superlattices.

To conform the general nature of the auxetic piezoelectric effect and demonstrate its thickness-limitation free nature, we systematically studied both (001)- and (111)-oriented [$SrTiO_3$/$Ba_{0.6}Sr_{0.4}TiO_3$/$BaTiO_3$] tricolour superlattice with a total thickness of about 100 nm (see **Fig. S13**). Each layer is set to have a nominal thickness of about 3 nm and their structures have been characterized by XRD measurements shown in **Fig. S14**a, b. The electrical properties of the superlattices have been detected by the capacitance vs voltage characteristics (see Fig. S14c, d). It indicates that there exists built-in potential of about 2.2 V and 0.86 V in (001)- and (111)-oriented superlattices, respectively. Such strong built-in fields induce substance electric polarization in the superlattices pointing from bottom electrode (i.e., Nb:$SrTiO_3$ substrate) to top electrode (i.e., Pt layer) and thus, give rise to the piezoelectric effect.

The piezoelectric property of the tricolour superlattices has been characterized using both the direct effect (i.e., applying stress and measuring short-circuit current) and the converse effect (i.e., applying voltage and measuring deformation/strain). As shown in **Fig. S15**, d_{33} and d_{31} of the (001)-oriented superlattice respectively show phase contrast of about $-90°$ and 90° with respect to the applied AC stress, indicating positive and negative signs of these two piezo-coefficients. Based on the linear fitting of the current density as function of the applied stress amplitude, we obtain the values of the piezoelectric coefficients as $d_{33} = 18.4\, pC/N$ and $d_{31} = -6.6\, pC/N$. In contrast, both d_{33} and d_{31} of the (111)-oriented superlattice exhibit phase values of about $-90°$. The piezo-coefficient of the (111)-superlattice is measured as $d_{33} = 6.6\, pC/N$ and $d_{31} = 3.3\, pC/N$, which corresponding to the auxetic piezoelectric effect.

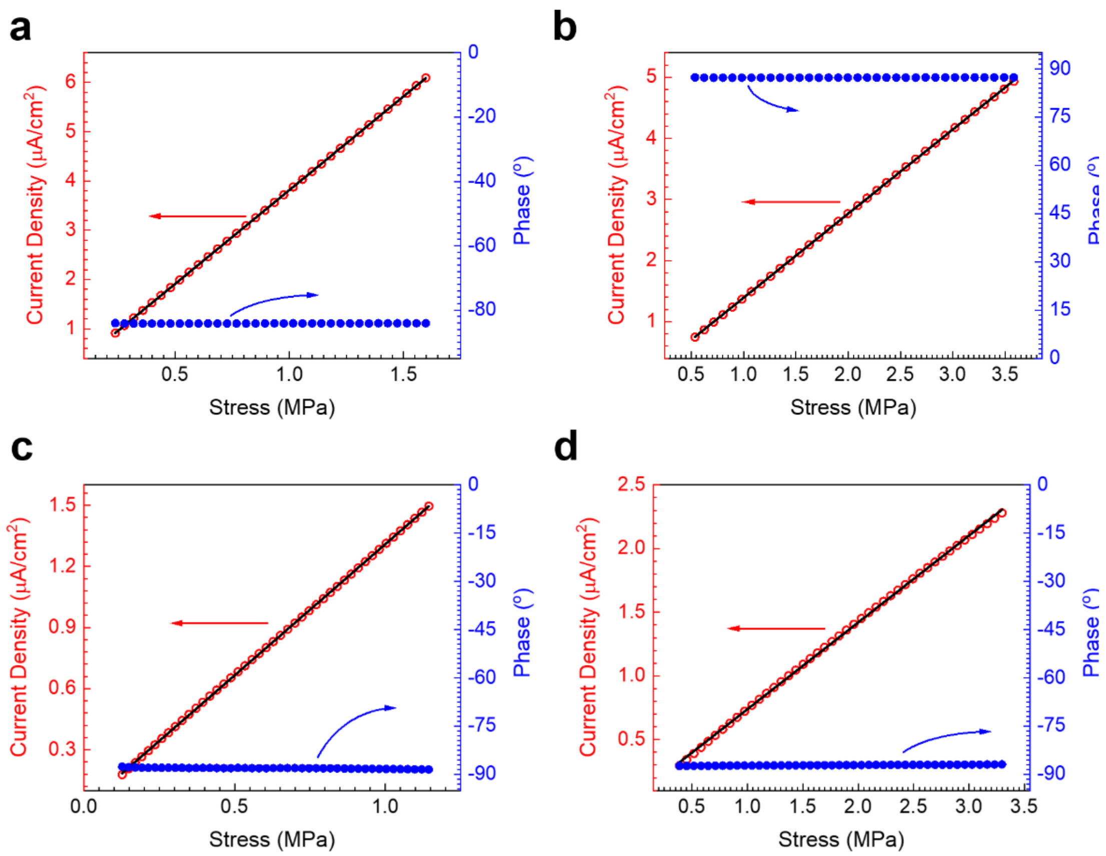

Fig. S15. Direct piezoelectric effect of [$SrTiO_3$/$Ba_{0.6}Sr_{0.4}TiO_3$/$BaTiO_3$]$_{10}$ superlattices. **a),** Longitudinal piezoelectric coefficient d_{33} characterization and **b)**, transverse piezo-coefficient d_{31} characterization of (001)-oriented superlattice. **c)**, d_{33} and **d)**, d_{31} measurements of (111)-oriented superlattice. The solid lines in the figures are the linear fitting of the current density as a function of the applied stress.

Note S11. In-plane orientation dependent piezoelectric coefficients

Rotating the in-plane axis x' and y' along the z'-axis in the newly oriented crystal will also modulate the form and value of the electrostriction tenor and piezoelectric coefficients. Among the three crystallographic orientations of interest, both (001)-and (111)-oriented $SrTiO_3$ crystals exhibit in-plane isotropic physical properties due to their C_4 and C_3 in-plane rotational symmetry. However, the (110)- oriented $SrTiO_3$ shows in-plane anisotropic properties due to its C_2 in-plane rotation symmetry. The electrostriction coefficients Q'_{3333}, Q'_{1133} and Q'_{2233} of (110)-oriented $SrTiO_3$ crystal by rotating the x' & y' along the z'-axis are given as

$$Q'_{3333} = \frac{1}{2}(Q^0_{11} + Q^0_{12} + 2Q^0_{44}) \quad \text{S13}$$

$$Q'_{1133} = \frac{1}{4}[Q^0_{11} + 3Q^0_{12} - 2Q^0_{44} + (-Q^0_{11} + Q^0_{12} + 2Q^0_{44})cos2\varphi] \quad \text{S14}$$

$$Q'_{2233} = \frac{1}{4}[Q^0_{11} + 3Q^0_{12} - 2Q^0_{44} + (Q^0_{11} - Q^0_{12} - 2Q^0_{44})cos2\varphi] \quad \text{S15}$$

where φ is the angle between the [110] crystallographic direction and the x'-axis that is rotated along the z'-axis. By taking the electrostriction coefficients Q_{11}, Q_{12}, Q_{44} into above equations, the variation of Q'_{1133}, Q'_{2233} and Q'_{3333} as functions of φ is shown in Fig. S8A. Q'_{3333} retains as a constant while rotating in-plane coordinate axis while Q'_{1133} and Q'_{2233} change in a cosine-style with a period of 180°. Q'_{1133} and Q'_{2233} are of opposite signs except at 45° & 135°, i.e., x'-axis is respectively parallel to the $[1\bar{1}\sqrt{2}]$ and $[\bar{1}1\sqrt{2}]$ crystallographic directions, where they converge. According to Eq.1 of the main text and using the values of the semiconducting properties of Nb:$SrTiO_3$ as used in the main text, we can obtain the angle φ dependent

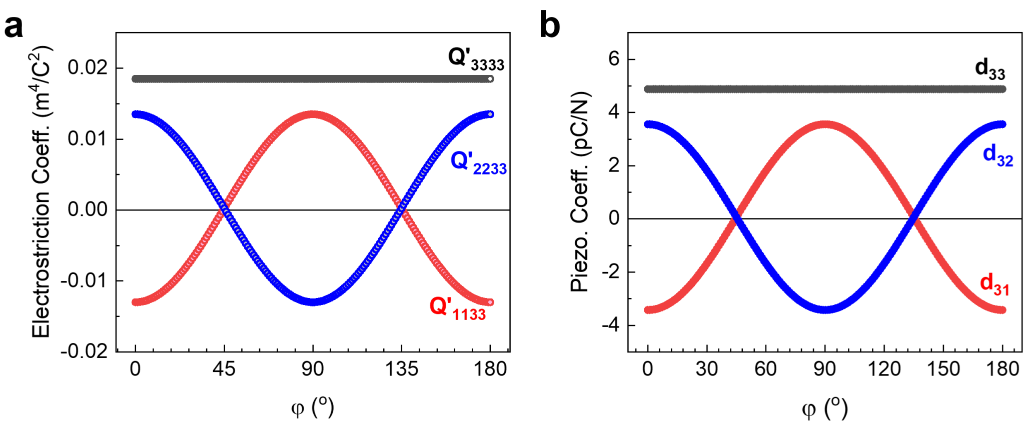

Fig. S16. Effect of rotating the x' & y' along the z'-axis on electromechanical properties of (110)-Au/Nb:$SrTiO_3$ junctions. a), Angle φ dependent electrostriction coefficients and **b**) variation of piezoelectric coefficients as functions of φ.

piezoelectric coefficients of the (110)-oriented Au/Nb:$SrTiO_3$ junction. As expected, d_{33} remains a constant while the transverse coefficients have a cosine-like dependency. The most interesting situations are at $45°$ & $135°$ where both d_{31} and d_{32} are zero. This means that (110)-Au/Nb:$SrTiO_3$ junction only possess longitudinal piezoelectric effect without any transverse effect if its side planes are set to $(1\bar{1}\sqrt{2})$ and $(\bar{1}1\sqrt{2})$, respectively.

Note S12: Preparation and characterization of Mo/4H-SiC junctions

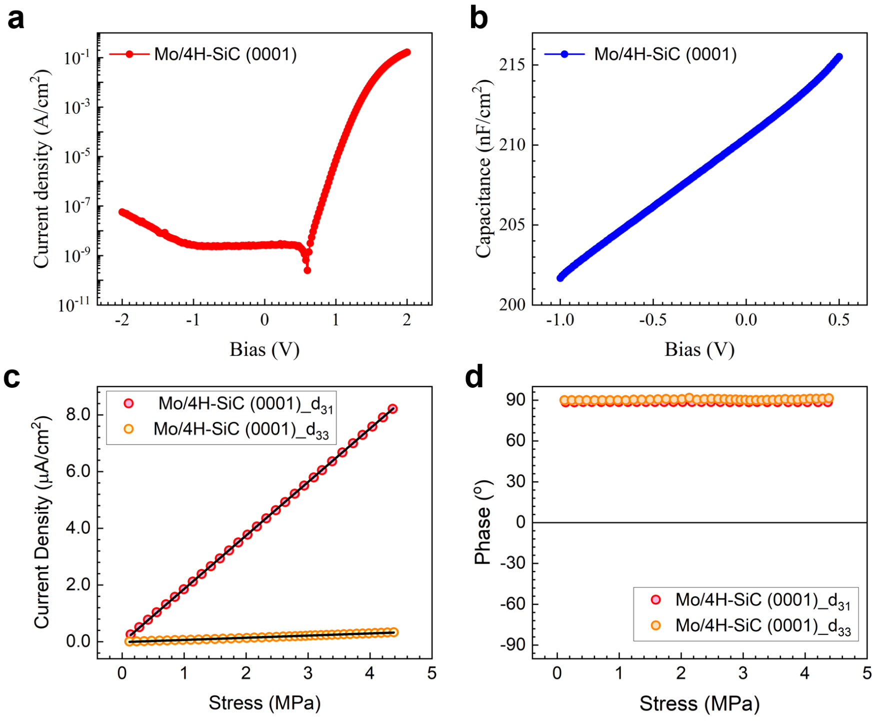

Fig. S17. Electrical and piezoelectric characterization of Mo/4H-SiC (0001) junctions. **a)**, Current-voltage curve. **b)**, Capacitance-voltage curve. **c)**, Current amplitude as function as the amplitude of applied stress. **d)**, Stress dependent phase.

Mo/4H-SiC planar Schottky diodes were fabricated on highly n-type (nitrogen-doped), 4° off-axis 4H-SiC substrates that had a 35 μm epitaxial layer (1x10^{15} cm^{-3} nitrogen-doped) grown on top of it. All chips underwent a standard RCA1 / HF (10%) / RCA2 / HF (10%) routine, after which the individual active device areas were defined and patterned using a conventional photolithography and mesa-isolation (dry etch) process. To assist the isolation between devices on each chip, a 1 μm thick silicon dioxide (SiO_2) layer was deposited by means of low-pressure chemical vapour deposition (LPCVD) using tetraethyl orthosilicate (TEOS) as Si precursor. This was then followed by the backside deposition of Ti (30nm) / Ni (100nm), leading to the ohmic contact formation after a rapid thermal anneal at 1000°C for 2 minutes in Ar (5 slm). The Schottky contacts were subsequently formed by opening up a window in the thick field oxide layer and evaporating 100 nm of Mo before annealing them at 500 °C in Ar 5 slm ambient. Processing was finished after a 1 μm thick Al metal overlay was evaporated on top of the device, serving as a field plate.

Note S13: The role of electrostriction coefficients on the auxetic piezoelectric effect

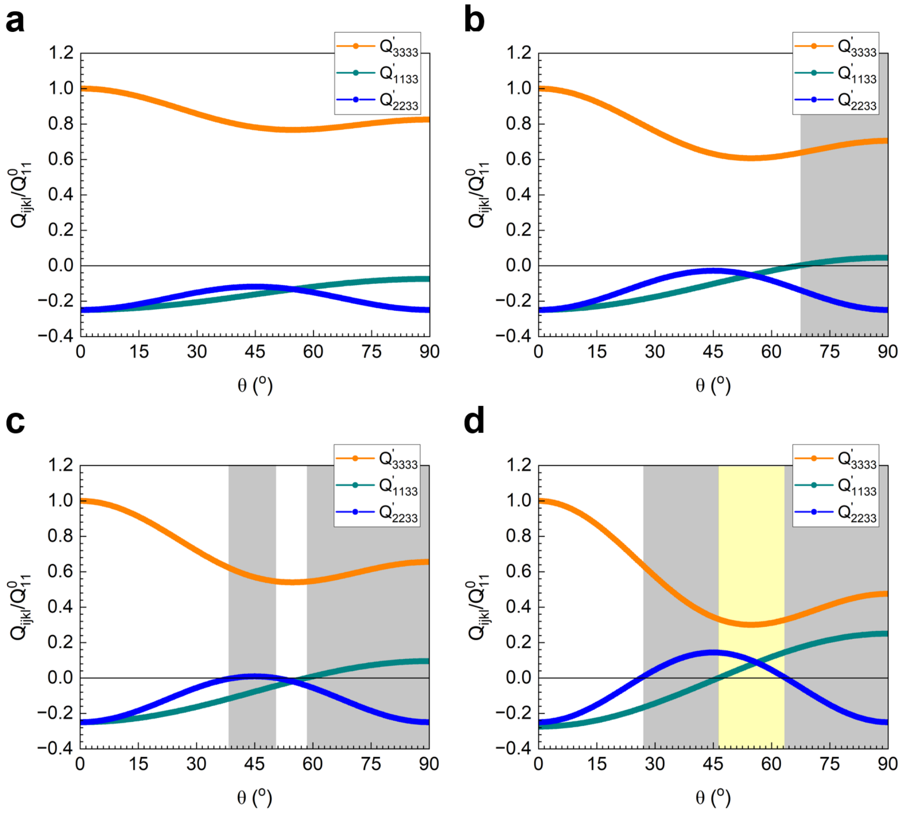

Fig. S18. Evolution of the electrostriction coefficients $\boldsymbol{Q_{ijkl}}$ by varying value $\boldsymbol{\eta}$. a), $\eta > (1+\nu)/2$. **b)**, $(3+5\nu)/6 < \eta < (1+\nu)/2$. **c)**, $(1+2\nu)/2 < \eta < (3+5\nu)/6$. **d)**, $\eta < (1+2\nu)/2$. Q'_{3333} always stays positive while the signs of Q'_{1133} and Q'_{2233} depend on the value of η. In **a)**, Q'_{3333} stay positive while Q'_{1133} and Q'_{2233} retain negative, indicating no Auxetic piezoelectric occurs. With decreasing the value of η, Q'_{1133} turns to positive as angle θ getting close to 90° as marked by the grey region in **b)**. With further reducing the value of η, Q'_{2233} becomes positive in the region close to 45°, as shown in **c)**. **b) & c)** correspond to the scenario that type-II auxetic piezoelectric effect can manifest. When η becomes smaller than $(1+2\nu)/2$, there appears a θ region where both Q'_{1133} and Q'_{2233} become positive, as marked by the yellow region in **d)**, which corresponds to the Auxetic piezoelectric effect.

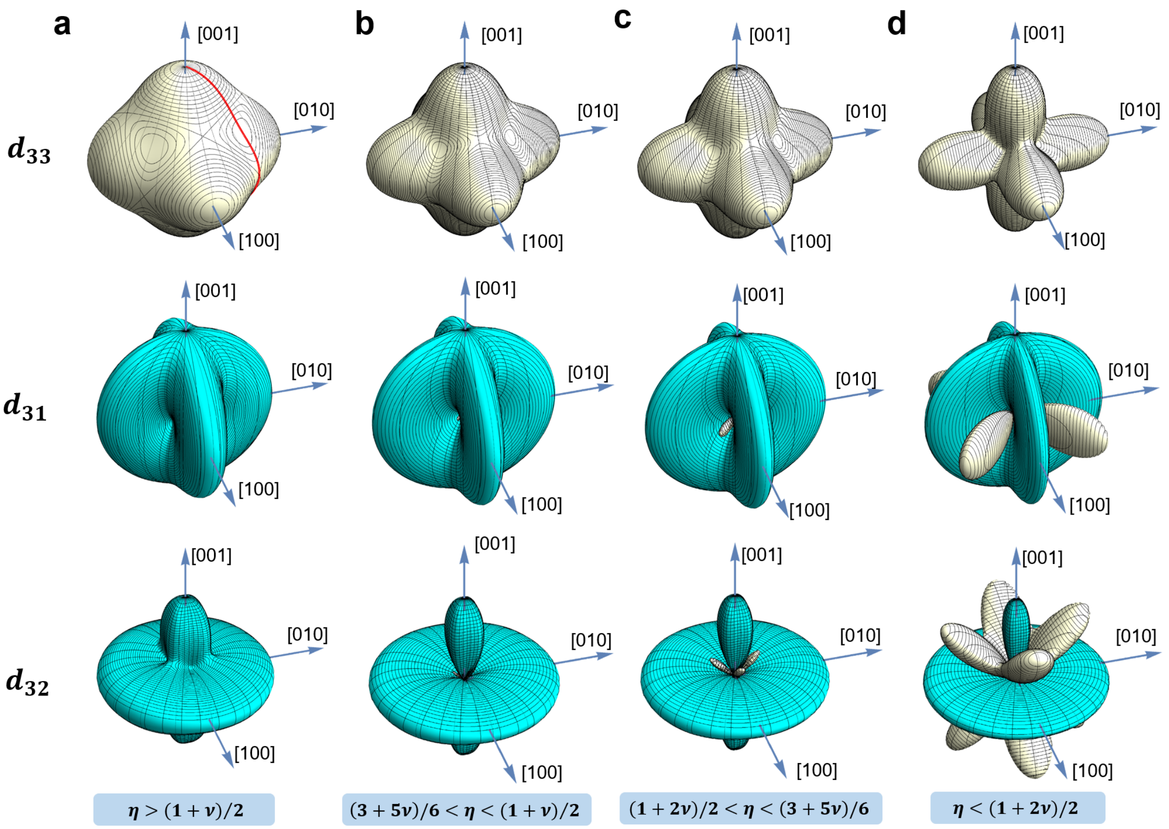

Fig. S19. The 3D-spherical plots of the piezoelectric coefficients d_{33}, d_{32} and d_{31} with varied η: **a**, $\eta > (1+\nu)/2$. **b**, $(3+5\nu)/6 < \eta < (1+\nu)/2$. **c**, $(1+2\nu)/2 < \eta < (3+5\nu)/6$. **d**, $\eta < (1+2\nu)/2$. The first column refers to the d_{33}, the second column refers to d_{32} and the last one corresponds to d_{31}. Clearly, with decreasing η, i.e., Q_{44}^{0}, the auxetic piezoelectric effect appears.

Note S14: Converse auxetic piezoelectric effect

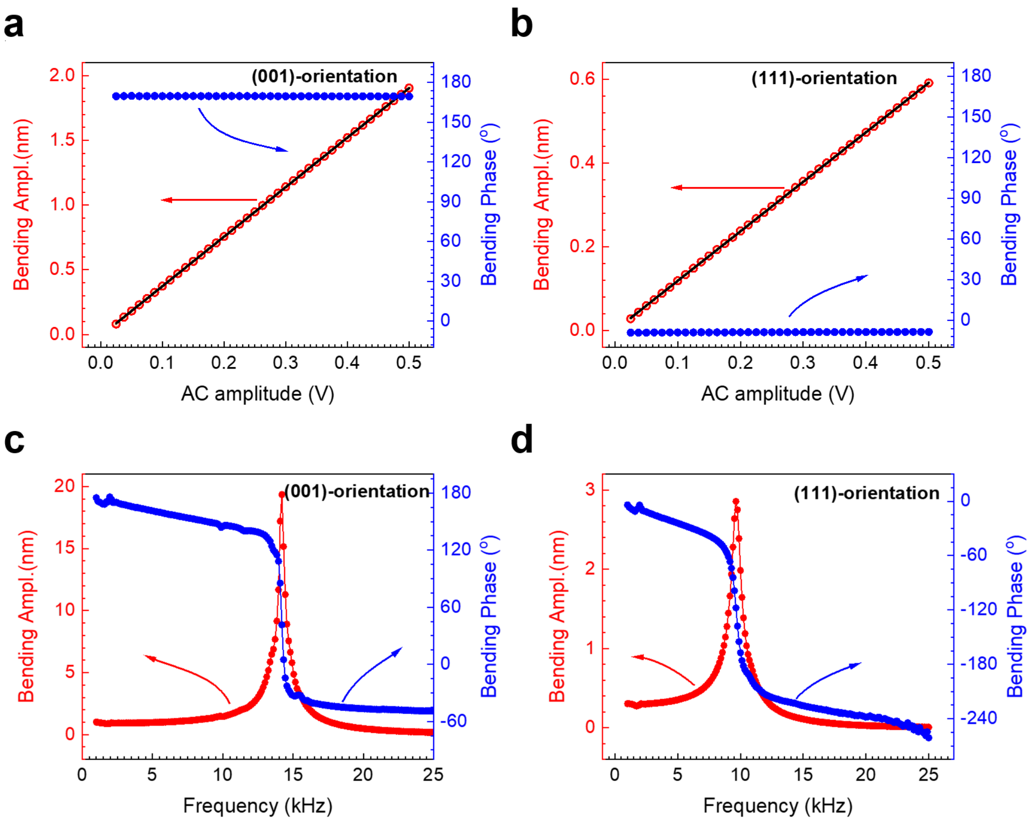

Fig. S20. AC voltage amplitude and frequency dependent superlattice cantilever vibration. The AC amplitude dependent bending amplitude (red dots) and phase (blue dots) of **a**), (001)-oriented and **b**), (111)-oriented cantilevers. The black curve is the linear fit. **c**), and **d**), show the frequency dependence of bending amplitude and phase. **c**) is the data of (001)-oriented device and **d**) corresponds to (111)-orientation.

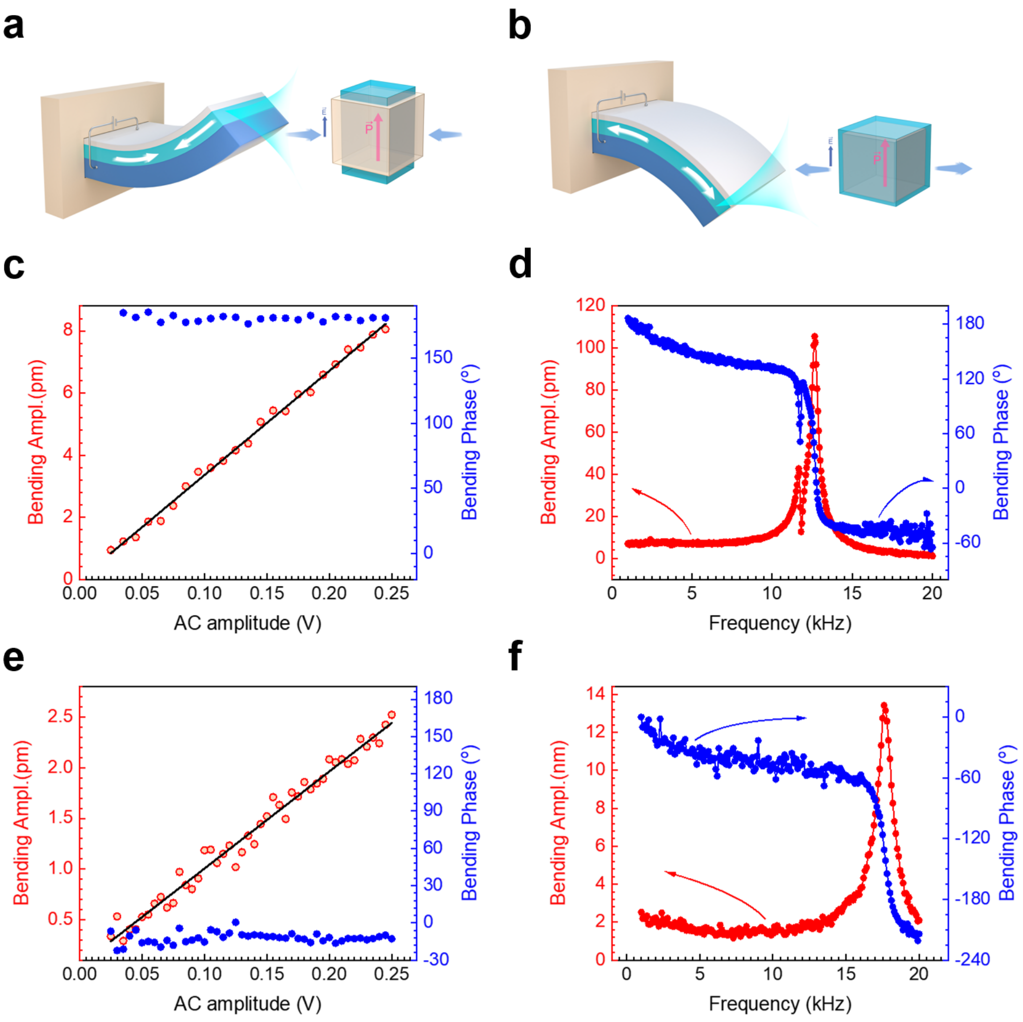

Fig. S21. Demonstration of the converse auxetic piezoelectric effect in Schottky junctions. a), Schematic shows the conventional piezoelectric effect induced cantilever bending. **b)**, Schematic shows the auxetic piezoelectric effect induced cantilever bending. The layer with white arrows refers to the piezoelectric active layer of the cantilever, which is the depletion region in the case of Schottky junctions. The dependence of bending amplitude and vibration phase of (001)-oriented Nb:$SrTiO_3$ junction cantilever on **c**) the amplitude of the 1.33kHz AC voltage and **d**) the frequency of AC voltage with an amplitude of 0.25 V. **e)** and **f)** respectively show the vibration dependence of the (111)-oriented Nb:$SrTiO_3$ junction cantilever on AC voltage amplitude and frequency. The AC frequency used in **e**) is 1.33kHz and the amplitude used in **f**) is 0.25 V.

References

S1 Haertling, G. H. Ferroelectric Ceramics: History and Technology. *Journal of the American Ceramic Society* **82**, 797-818(1999).

S2 Warner, A. W., Coquin, G. A. & Fink, J. L. Elastic and Piezoelectric Constants of $Ba_2NaNb_5O_{15}$. *Journal of Applied Physics* **40**, 4353-4356(1969).

S3 Wen, Y. *et al.* Growth and electrical properties of lead-free ferroelectric single crystal $Ba_{0.77}Ca_{0.23}TiO_3$. *Ceramics International* **48**, 25628-25636(2022).

S4 Xie, L., Hou, S., Yu, F. & Zhao, X. Single crystal growth and piezoelectric features of the $Ca_2Nb_2O_7$ crystal with orthorhombic symmetry. *CrystEngComm* **24**, 8117-8125(2022).

S5 Liminga, R., Abrahams, S. & Bernstein, J. Absolute sense and model for the piezoelectric and pyroelectric coefficients in $Ba(NO_2)_2H_2O$ and $Cs_2S_2O_6$. *Journal of Applied Crystallography* **13**, 516-520(1980).

S6 Ishikawa, M., Kadota, Y., Takiguchi, N., Hosaka, H. & Morita, T. Synthesis of Nondoped Potassium Niobate Ceramics by Ultrasonic Assisted Hydrothermal Method. *Japanese Journal of Applied Physics* **47**, 7673-7677(2008).

S7 Warner, A. W., Onoe, M. & Coquin, G. A. Determination of Elastic and Piezoelectric Constants for Crystals in Class (3m). *The Journal of the Acoustical Society of America* **42**, 1223-1231(1967).

S8 Jung, H.-R., Jin, B.-M., Cha, J.-W. & Kim, J.-N. Piezoelectric and elastic properties of $Li_2B_4O_7$ single crystal. *Materials Letters* **30**, 41-45(1997).

S9 Ikeda, T. & Imazu, I. Piezoelectric study of Li_2GeO_3 crystal. *Japanese Journal of Applied Physics* **15**, 1451(1976).

S10 Haussühl, S., Liebertz, J. & Stähr, S. Single crystal growth and pyroelectric, dielectric, piezoelectric, elastic, and thermoelastic properties of orthorhombic Li_2SiO_3, Li_2GeO_3, and Na_2GeO_3. *Crystal Research and Technology* **17**, 521-526(1982).

S11 Li, Z., Grimsditch, M., Xu, X. & Chan, S. K. The elastic, piezoelectric and dielectric constants of tetragonal $PbTiO_3$ single crystals. *Ferroelectrics* **141**, 313-325(2011).

S12 Nomura, S. & Uchino, K. Electrostrictive effect in $Pb(Mg_{1/3}Nb_{2/3})O_3$-type materials. *Ferroelectrics* **41**, 117-132(2011).

S13 Hayward, G., Bennett, J. & Hamilton, R. A theoretical study on the influence of some constituent material properties on the behavior of 1 - 3 connectivity composite transducers. *The Journal of the Acoustical Society of America* **98**, 2187-2196(1995).

S14 Qiao, L. *et al.* Temperature Dependence of Elastic, Piezoelectric, and Dielectric Matrixes of [001]-Poled Rhombohedral PIN-PMN-PT Single Crystals. *IEEE Trans Ultrason Ferroelectr Freq Control* **66**, 1786-1792(2019).

S15 Yang, S. *et al.* Full matrix electromechanical properties of textured $Pb(In_{1/2}Nb_{1/2})O_3$-$Pb(Sc_{1/2}Nb_{1/2})O_3$-$PbTiO_3$ ceramic. *Journal of Applied Physics* **131**, 124104(2022).

S16 Poterala, S. F., Trolier-McKinstry, S., Meyer, R. J. & Messing, G. L. Processing, texture quality, and piezoelectric properties of <001> Ctextured (1-x)$Pb(Mg_{1/3}Nb_{2/3})TiO_3$-x$PbTiO_3$ceramics. *Journal of Applied Physics* **110**, 014105 (2011).

S17 Ogawa, T., Yamauchi, Y., Numamoto, Y., Matsushita, M. & Tachi, Y. Giant Electromechanical Coupling Factor of k_{31} Mode and Piezoelectric d_{31} Constant in $Pb[(Zn_{1/3}Nb_{2/3})_{0.91}Ti_{0.09}]O_3$ Piezoelectric Single Crystal. *Japanese Journal of Applied Physics* **41**, L55-L57(2002).

S18 Huang, L. *et al.* Large and temperature-independent piezoelectric response in $Pb(Mg_{1/3}Nb_{2/3})O_3$-$BaTiO_3$-$PbTiO_3$. *Applied Physics Letters* **101**, 192901(2012).

S19 Yu, F., Hou, S., Zhang, S., Lu, Q. & Zhao, X. Electro-elastic properties of $YCa_4O(BO_3)_3$ piezoelectric crystals. *physica status solidi (a)* **211**, 574-579(2014).

S20 Ye, S., Fuh, J., Lu, L., Chang, Y.-l. & Yang, J.-R. Structure and properties of hot-pressed lead-free $(Ba_{0.85}Ca_{0.15})(Zr_{0.1}Ti_{0.9})O_3$ piezoelectric ceramics. *RSC Advances* **3**, 20693-20698 (2013).

S21 Gao, W. L. *et al.* Growth, electromechanical, and electro-optic properties of tungsten bronze $(Ca_{0.28}Ba_{0.72})_{0.25}(Sr_{0.6}Ba_{0.4})_{0.75}Nb_2O6$ single crystal. *Journal of Applied Physics* **107**, 094101 (2010).

S22 Chang, Y., Poterala, S. F., Yang, Z., Trolier-McKinstry, S. & Messing, G. L. ⟨001⟩ textured $(K_{0.5}Na_{0.5})(Nb_{0.97}Sb_{0.03})O_3$ piezoelectric ceramics with high electromechanical coupling over a broad temperature range. *Applied Physics Letters* **95**, 232905(2009).

S23 Zhang, R., Jiang, B., Jiang, W. & Cao, W. Anisotropy in domain engineered 0.92 $Pb(Zn_{1/3}Nb_{2/3})O_3$-0.08$PbTiO_3$ single crystal and analysis of its property fluctuations. *IEEE Transactions on Ultrasonics, Ferroelectrics, and Frequency Control* **49**, 1622-1627(2002).

S24 Jiang, Y. *et al.* Microstructure, dielectric, and piezoelectric properties of 0.38$Bi(Ga_xSc_{1-x})O_3$–0.62$PbTiO_3$ high temperature piezoelectric ceramics. *physica status solidi (RRL) – Rapid Research Letters* **2**, 28-30(2008).

S25 Austerman, S. B., Berlincourt, D. A. & Krueger, H. H. A. Polar Properties of BeO Single Crystals. *Journal of Applied Physics* **34**, 339-341(1963).

S26 Crisler, D., Cupal, J. & Moore, A. Dielectric, piezoelectric, and electromechanical coupling constants of zinc oxide crystals. *Proceedings of the IEEE* **56**, 225-226(1968).

S27 Fjeldly, T. A. & Hanson, R. C. Elastic and piezoelectric contants of silver-iodide: Study of a material at the covalent-ionic phase transition. *Physical Review B* **10**, 3569-3577(1974).

S28 Tsubouchi, K. & Mikoshiba, N. Zero-temperature-coefficient SAW devices on AlN epitaxial films. *IEEE Transactions on Sonics Ultrasonics* **32**, 634-644(1985).

S29 Zhang, H. *et al.* Process Control Monitor (PCM) for Simultaneous Determination of the Piezoelectric Coefficients d_{31} and d_{33} of AlN and AlScN Thin Films. *Micromachines (Basel)* **13**, 581(2022).

S30 Assali, A., Laidoudi, F., Serhane, R., Kanouni, F. & Mezilet, O. Highly Enhanced Electro-acoustic Properties of YAlN/Sapphire Based Surface Acoustic Wave Devices for Next Generation of Microelectromechanical Systems. *Materials Today Communications* **26**, 102067(2021).

S31 Joffe, H., Berlincourt, D., Krueger, H. & Shiozawa, L.Piezoelectric properties of cadmium sulfide crystals. in *14th Annual Symposium on Frequency Control*, 19-23(1960).

S32 Berlincourt, D., Jaffe, H. & Shiozawa, L. R. Electroelastic Properties of the Sulfides, Selenides, and Tellurides of Zinc and Cadmium. *Physical Review* **129**, 1009-1017(1963).

S33 Guy, I. L., Muensit, S. & Goldys, E. M. Extensional piezoelectric coefficients of gallium nitride and aluminum nitride. *Applied Physics Letters* **75**, 4133-4135(1999).

S34 Jean-Mistral, C., Basrour, S. & Chaillout, J. J. Comparison of electroactive polymers for energy scavenging applications. *Smart Materials and Structures* **19**, 085012(2010).

S35 Omote, K., Ohigashi, H. & Koga, K. Temperature dependence of elastic, dielectric, and piezoelectric properties of "single crystalline'' films of vinylidene fluoride trifluoroethylene copolymer. *Journal of Applied Physics* **81**, 2760-2769(1997).

S36 Abbasipour, M., Khajavi, R. & Akbarzadeh, A. H. A Comprehensive Review on Piezoelectric Polymeric and Ceramic Nanogenerators. *Advanced Engineering Materials* **24**, 2101312(2022).

S37 W. G. Cady, *Piezoelectricity: Volume Two: An Introduction to the Theory and Applications of Electromechanical Phenomena in Crystals*. (Courier Dover Publications, 2018).

S38 X.-h. Du, Q.-M. Wang, U. Belegundu, A. Bhalla, K. Uchino, Crystal orientation dependence of piezoelectric properties of single crystal barium titanate. *Materials Letters* **40**, 109-113 (1999).

S39 J. Gao, D. Xue, W. Liu, C. Zhou, X. Ren, in *Actuators*. (MDPI, 2017), vol. 6, pp. 24.

S40 M.-M. Yang, Z.-D. Luo, Z. Mi, J. Zhao, M. Alexe, Piezoelectric and pyroelectric effects induced by interface polar symmetry. *Nature* **584**, 377-381 (2020).

S41 E. Mikheev, B. D. Hoskins, D. B. Strukov, S. Stemmer, Resistive switching and its suppression in Pt/Nb: $SrTiO_3$ junctions. *Nature Communications* **5**, 3990 (2014).

S42 P. E. Blöchl, Projector augmented-wave method. *Physical Review B* **50**, 17953 (1994).

S43 G. Kresse, D. Joubert, From ultrasoft pseudopotentials to the projector augmented-wave method. *Physical Review B* **59**, 1758 (1999).

S44 J. P. Perdew *et al.*, Restoring the density-gradient expansion for exchange in solids and surfaces. *Physical Review Letters* **100**, 136406 (2008).

S45 A. Biswas *et al.*, Universal Ti-rich termination of atomically flat SrTiO 3 (001),(110), and (111) surfaces. *Applied Physics Letters* **98**, 051904 (2011).

S46 R. King-Smith, D. Vanderbilt, Theory of polarization of crystalline solids. *Physical Review B* **47**, 1651 (1993).

S47 D. Vanderbilt, R. King-Smith, Electric polarization as a bulk quantity and its relation to surface charge. *Physical Review B* **48**, 4442 (1993).

S48 W. Tang, E. Sanville, G. Henkelman, A grid-based Bader analysis algorithm without lattice bias. *Journal of Physics: Condensed Matter* **21**, 084204 (2009).
